# The dynamic architecture of Map1- and NatB-ribosome complexes coordinates the sequential modifications of nascent polypeptide chains

**DOI:** 10.1371/journal.pbio.3001995

**Published:** 2023-04-20

**Authors:** Alexandra G. Knorr, Timur Mackens-Kiani, Joanna Musial, Otto Berninghausen, Thomas Becker, Birgitta Beatrix, Roland Beckmann

**Affiliations:** Department of Biochemistry, Gene Center, Ludwig-Maximilians University Munich, University of Munich, Munich, Germany; University of California, Berkeley, UNITED STATES

## Abstract

Cotranslational modification of the nascent polypeptide chain is one of the first events during the birth of a new protein. In eukaryotes, methionine aminopeptidases (MetAPs) cleave off the starter methionine, whereas N-acetyl-transferases (NATs) catalyze N-terminal acetylation. MetAPs and NATs compete with other cotranslationally acting chaperones, such as ribosome-associated complex (RAC), protein targeting and translocation factors (SRP and Sec61) for binding sites at the ribosomal tunnel exit. Yet, whereas well-resolved structures for ribosome-bound RAC, SRP and Sec61, are available, structural information on the mode of ribosome interaction of eukaryotic MetAPs or of the five cotranslationally active NATs is only available for NatA. Here, we present cryo-EM structures of yeast Map1 and NatB bound to ribosome-nascent chain complexes. Map1 is mainly associated with the dynamic rRNA expansion segment ES27a, thereby kept at an ideal position below the tunnel exit to act on the emerging substrate nascent chain. For NatB, we observe two copies of the NatB complex. NatB-1 binds directly below the tunnel exit, again involving ES27a, and NatB-2 is located below the second universal adapter site (eL31 and uL22). The binding mode of the two NatB complexes on the ribosome differs but overlaps with that of NatA and Map1, implying that NatB binds exclusively to the tunnel exit. We further observe that ES27a adopts distinct conformations when bound to NatA, NatB, or Map1, together suggesting a contribution to the coordination of a sequential activity of these factors on the emerging nascent chain at the ribosomal exit tunnel.

## Abstract 2

Cryo-EM structures of yeast methionine aminopeptidase and N-acetyltransferase B in complex with ribosomes shed light on the involvement of rRNA expansion segment ES27a in orchestrating modifications of newly emerging polypeptide chains during translation.

## Introduction

In all kingdoms of life, nascent polypeptide chains are subject to chemical modification as soon as they emerge from the ribosomal exit tunnel. The earliest and most common modifications in eukaryotes are the cleavage of the N-terminal amino acid and Nα-acetylation, both of which can play important roles in the targeting, folding, and stability of the newly made polypeptide.

Translation of the vast majority of mRNAs starts on an AUG codon with a methionine-bound initiator tRNA (Met-tRNAi-Met), resulting in methionine as the first amino acid of most proteins. When this starter methionine is followed by a small and uncharged amino acid, such as Ala, Cys, Gly, Pro, Ser, Thr, or Val, it is usually removed cotranslationally by evolutionarily conserved methionine aminopeptidases (MetAPs) [1–3]. There are two types of MetAPs. While type I is found in eubacteria and type II in archaea, eukaryotes harbor both types of MetAPs. The two types differ in a characteristic insertion (approximately 60 aa) in the catalytic domain of type II enzymes [4]. The common catalytic domain belongs to the family of evolutionarily conserved metalloproteases and adopts the typical aminopeptidase fold also known as “pita-bread” protease fold [5]. Catalysis typically requires one or two divalent cations (for review, see [6,7]). In contrast to bacterial MetAPs, eukaryotic MetAP1s possess an additional N-terminal extension containing two zinc finger domains, a RING-finger-like Cys2-Cys2 zinc finger (aas 22 to 40 in yeast) and a Cys2-His2 zinc finger (aas 50 to 66 in yeast) related to RNA-binding zinc fingers. This extension has been suggested to be important for the correct functional alignment of Map1 on the ribosome in yeast [8]. Eukaryotic type II MetAPs also contain an N-terminal extension carrying a positively charged Lys-rich stretch [7,9].

The fundamental importance of N-terminal methionine removal is reflected by the lethality caused by deletion of all MetAP-encoding genes in eubacteria [10,11] and yeast [4]. In baker’s yeast (*Saccharomyces cerevisiae; S*. *cerevisiae*), Map1 (a type I MetAP) represents the major isoform, indicated by the higher copy number as well as by a much stronger slow growth phenotype of *map1* null mutants when compared to *map2* null mutants [12–14]. Both Map1 and Map2 were previously shown to bind to ribosomes [8,15], and the ribosome interaction of Map1 was shown to be salt-sensitive and independent of the nascent polypeptide chain. Moreover, the Map1-interacting region was mapped via cross-linking studies to the peptide exit tunnel periphery of the 60S subunit, likely contacting the region covered by uL23 and uL29 [16]. This position is overlapping with contact sites of various exit tunnel-binding factors such as the chaperones RAC (ribosome-associated complex) and NAC (nascent polypeptide-associated complex), as well as the secretory pathway factors SRP (signal recognition particle) and the Sec61 protein-conducting channel. Furthermore, evidence was provided for an involvement of rRNA expansion segment ES27 in the interaction of MAPs with the ribosome since deletion of the longest helix of ES27 resulted in a decrease of Map1 and Map2 ribosome association in *S*. *cerevisiae* [17,18].

For unmodified nascent peptides but also for nascent peptides after methionine cleavage, Nα-acetylation is another highly frequent cotranslational modification in eukaryotes. It is catalyzed by Nα-acetyltransferases (NATs), which transfer an acetyl group from acetyl-coenzyme A (acetyl-CoA) to the α-amino group of the emerging nascent chain. With the exception of NatD, ribosome-associated NATs form dimeric or trimeric hetero-complexes and usually consist of a small catalytic and at least one additional large auxiliary subunit [19]. In humans, seven subtypes of NATs exist: NatA to NatH, with the first three of them, NatA, NatB, and NatC acetylating the majority of substrate proteins [19–21]. The most abundant member of the Nα-acetyltransferase family is NatA, which modifies nascent chains with an N-terminal Ser, Ala, Thr, Gly, Val, or Cys after the initiator methionine has been removed by MetAPs. In contrast, Nα-acetylation by NatB and NatC occurs without initiator methionine removal, since they directly acetylate this methionine when followed by Asp, Glu, Asn, or Gln (in case of NatB) or by large hydrophobic residues including Leu, Ile, Phe, and Tyr (in case of NatC).

In yeast, it was shown that whereas N-α-acetylation by NatA seems to play a role in systemic adaptation control, modification by NatB seems rather to be important for protein folding [22]. Furthermore, depletion of NatB subunits to 30% of the wild-type level caused a 50% decrease in growth in *Arabipopsis thaliana* [23] and missense mutations in the catalytic domain NAA20 of human NatB were shown to cause autosomal recessive developmental delay, intellectual disability, and microcephaly, emphasizing the importance of NatB function for the cell [24].

Given the differences in substrate specificity and in requirement for initiator methionine removal, the question arises how access of MetAPs and the different NATs to the peptide exit site is spatially and temporally coordinated. Here, a recent cryo-EM structure of yeast NatA bound to native 80S ribosomes carrying a nascent chain showed that NatA is anchored directly at the exit tunnel by interactions with ribosomal rRNA expansion segments (ESs) [25]. NatA was found in a position sterically excluding concomitant binding of other cotranslationally acting chaperones (RAC/Ssb), SRP, as well as Sec61. Also recently, the structures of NatB from *Candida albicans* [26] and human NatB [27] as well as *Chaetomium thermophilum* Naa20 with a competitive inhibitor [28] were determined by X-ray crystallography and cryo-EM, respectively. However, for eukaryotic MAPs or the other NATs, structural information on their mode of ribosome interaction is largely lacking and the interplay of these factors at the exit tunnel is thus only poorly understood.

Here, we present cryo-EM structures of nascent chain-carrying 80S ribosomes in complex with Map1 or the NatB complex from *S*. *cerevisiae* at an overall resolution of 3.8 to 3.9 Å (for Map1-80S) and 3.1 to 3.8 Å (for NatB-80S), respectively. We observed Map1 mainly flexibly bound to the dynamic rRNA expansion segment ES27a that positions it directly juxtaposed the peptide exit tunnel. This position would allow for a very early encounter with substrate nascent chains and explain their modification as soon as they emerge from the ribosome tunnel. Moreover, the observed binding mode of Map1 likely excludes concomitant NatA binding for subsequent Nα-acetylation of the new N-terminus.

Despite also being recruited to the peptide tunnel exit periphery, NatB shows a substantially different binding mode at the exit site compared to NatA. Interestingly, we observe two copies of NatB, one bound directly below the tunnel exit site and to ES27a (NatB-1) and one bound to the second universal adapter site (UAS-II) for exit factors (NatB-2) [29]. In contrast to NatA, NatB-2 engages also via contacts to rigid parts of rRNA and the ribosomal protein eL31. For both NatBs, the catalytic subunits are positioned below the tunnel exit such that nascent chains could engage them at a length of approximately 55 amino acids. Yet, we speculate that NatB-2 is more likely to engage the substrate, while NatB-1 might serve to position NatB-2 via ES27a.

## Results

### Cryo-EM structure of the Map1-ribosome complex

**Fig 1:**
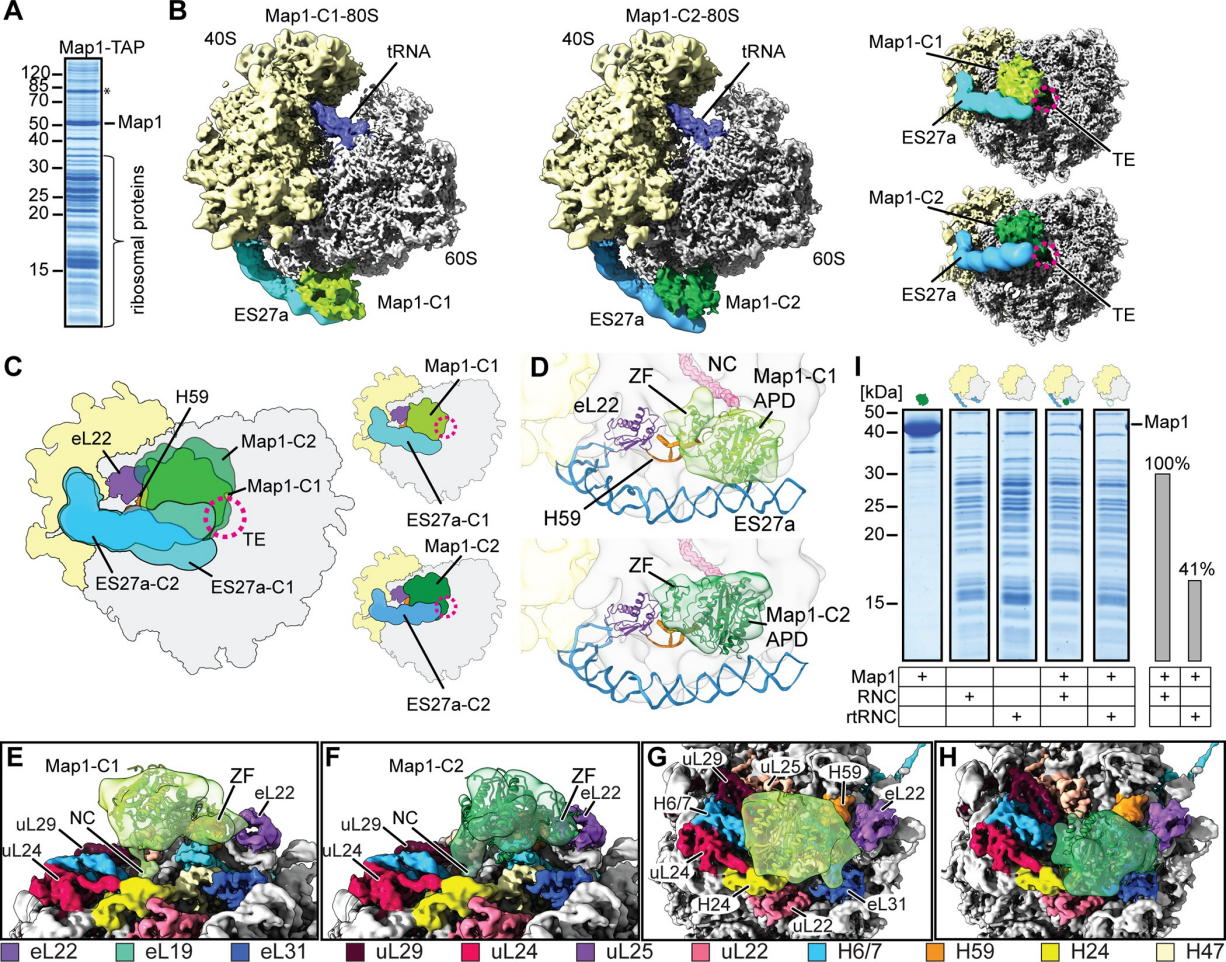
Cryo-EM structures of Map1 bound to ribosomes via expansion segment ES27a. (**A**) Concentrated eluate obtained from Map1-TAP affinity purification shown on a 12% Nu-PAGE. Mass spectrometry analysis confirmed the presence of Map1 and ribosomal proteins. A contamination from a viral protein is marked with an asterisk. See S1 Raw Images for the raw gel image. (**B**) Cryo-EM structures of Map1 in complex with a translating 80S ribosome in two different conformations (left: Map1-C1-80S; center: Map1-C2-80S; right: bottom view on the peptide exit tunnel). The maps were filtered according to local resolution. Isolated densities were extracted after the final CTF refinement. (**C**) Cartoon representation of bottom views with overlay (left) and separate views (right) as shown in (**B**). (**D**) An AlphaFold 2 model for Map1 was fitted into the density, shown as front view. The maps were filtered to 20 Å using a Gaussian low-pass filter. (**E**-**H**) Two views on the tunnel exit highlighting the position of Map1-C1 (**E**, **G**) and Map1-C2 (**F**, **H**) with respect to tunnel exit surrounding ribosomal proteins coloured as indicated in the legend below. (**I**) In vitro binding assay addressing the contribution of ES27a to ribosome-Map1 binding. Samples from the pelleting assay using recombinant Map1 and purified RNaseI-treated (rtRNC) or nontreated RNCs were applied to a 15% SDS-PAGE. For Map1 alone the supernatant (SN) fraction and for all other samples the pellet (P) fraction is shown. Co-pelleting of Map1 with the ribosome was quantified by densitometry. When ESs were digested by RNAseI, Map1 binding was significantly decreased to about 40% compared to Map1 binding to untreated 80S ribosomes. TE: tunnel exit. ZF: zinc finger domain. APD: aminopeptidase domain. Map1-C1: light green, Map1-C2: dark green, eL22: purple, H59: orange, 40S SU: light yellow, 60S SU: grey, ES27a: cyan, tRNAs: dark blue, nascent chain (NC): pink. See S1 Raw Images for raw gel images and S1 Data for numerical data underlying the quantification.

Cryo-EM samples for endogenous MetAP-ribosome complexes were obtained from native pullouts of ribosome-bound TAP-tagged Map1 essentially as described before [25,30] (Figs 1A and S1). After elution by cleavage of the Map1-tag using tobacco etch virus (TEV) protease, the Map1-ribosome complexes were stabilized by treatment with the chemical crosslinker glutaraldehyde prior to cryo-grid preparation. As observed before in native pullouts with cycloheximide-treated samples [25,30], 3D classification revealed classes with programmed ribosomes predominantly in the pre-translocational state with tRNAs present in the canonical A and P sites, but also classes representing termination/pre-recycling complexes (with eRF1 and ABCE1) and idle (tRNA-free) ribosomes (S2 Fig). Notably, in the majority of classes, ES27a was found in the position below the peptide exit tunnel (ES27-exit), and attached to ES27a, we observed an additional density reaching to the peptide exit tunnel. We joined those classes and subjected them to focused 3D classification using a soft mask for the exit tunnel/ES27a region. Two classes were enriched, showing a prominent globular, nonribosomal extra density attached to ES27a in different conformations below the peptide exit. These two classes differed mainly in the position of ES27a and its attached nonribosomal density, but not in the overall ribosomal state. Although the additional density could not be better resolved due to its flexibility, based on its position, overall shape, and dimension, we assigned it to Map1 (Figs 1B, 1C, and S2). ES27a in the exit position serving as the principal binding site for Map1 is in agreement with deletion experiments where the tip of ES27a was shortened, leading to reduced levels of ribosome associated Map1 [17,18]. Furthermore, our assignment agrees with biochemical findings based on chemical cross-linking, showing Map1 close to uL29, a protein located adjacent to the tunnel exit [16].

While both classes could be refined to an overall resolution of 3.8 and 3.9 Å (referred to as Map1-C1-80S and Map1-C2-80S), respectively, local resolution of Map1 bound to ES27a was limited to 7 Å and below (S3 Fig). This indicates a high degree of flexibility of the Map1-ribosome interaction, most likely owing in part to the flexibility of its binding partner ES27a, which can cover a continuous conformational space between tunnel exit site and L1 protuberance [31]. However, since local refinement attempts using the multibody approach in RELION also failed, we concluded that the flexibility of ES27a does not solely prevent higher resolution but that the binding of Map1 itself to ES27a is flexible. Accordingly, we were not able to gain higher local resolution required to build a molecular model for the regions comprising ES27a and Map1.

In both classes, the position of Map1 at the exit tunnel is similar to that of the homologs of Map1 involved in 60S subunit biogenesis, yeast Arx1 (associated with ribosomal export complex protein 1) and human EBP1 (ErbB3-binding protein 1) [32–35]. Moreover, Map1-C1 superimposes well with the bacterial Map visualized in a PDF-Map-70S ribosome complex from *Escherichia coli* [36] (S4 Fig).

Thus, despite the rather low resolution, the reconstructions allowed us to fit a model of Map1 generated by AlphaFold 2 (AF2) [37] into the respective densities, thereby providing an idea of the overall positioning of Map1 with respect to the ribosome (Fig 1D). The fits were guided by high-resolution cryo-EM structures of ribosome-bound EBP1 [34,35,38] (S4 Fig). After fitting the human 80S-EBP1 models into our densities for an overall orientation determination of Map1, we superimposed the AF2 model for Map1 and rigid body fitted it separately into isolated densities (Figs 1D and S5A–S5D). This resulted in positioning of the globular amino peptidase domain (APD) of Map1 below the peptide exit tunnel contacting ES27a and H59. Density for the APD spans from UAS II (comprising eL31 and eL22) to UAS I (comprising uL23 and uL29) (Fig 1E–1H) [29] and is thus in agreement with published cross-linking data [16]. In addition, density for the nascent chain was visible, representing a broad variety in composition and lengths of nascent chains obtained through the native pullout. Therefore, a possible influence of differences in the nature and length of the nascent chain on the two observed Map1 states cannot be addressed.

In addition to the large APD, AF2 also predicts the structure of the two zinc finger (ZF) domains of Map1 at a position that coincides with extra density observed in our maps, locating the zinc fingers adjacent to ribosomal protein eL22 (Fig 1D–1F).

As stated above, ES27a is the main contact site for Map1 to the 60S ribosomal subunit. ES27 consists of three A helices, which can change their position flexibly around the three-way junction connecting rRNA helices H63, ES27a, and ES27b (nomenclature according to Petrov and colleagues [39]). The longest helix, ES27a, thereby undergoes the most severe conformational changes. In yeast, so far, two main positions are known, one with the tip of ES27a facing towards the L1 stalk of the 60S (L1-position) and one facing towards the peptide exit tunnel (exit-position) [31]. Interestingly, we observed ES27a-exit in two novel stabilized conformations, when bound to Map1. Compared to ES27a-bound NatA complexes, in Map1 complexes, ES27a is rotated with the three-way junction as a pivot by 31 degrees for conformation 1 and by 19 degrees for conformation 2 (S6 Fig).

To confirm the major contribution of ES27a to Map1 ribosome binding, we performed in vitro binding assays with purified Map1 and ribosome nascent chain complexes (RNCs) or RNaseI-treated RNCs (rtRNCs), as done before for NatA [25]. In rtRNCs, rRNA ESs are clipped off by the RNAseI treatment, as previously shown [25]. Map1 binding to rtRNCs was significantly reduced by about 60% when comparing to untreated RNCs, again confirming that ES27a is a major binding site for Map1 recruitment to the ribosomal exit site (Fig 1I).

Taken together, our analysis shows that Map1 is bound to the 80S ribosome in the immediate vicinity of the ribosomal tunnel exit mainly via a flexible association with the dynamic rRNA ES27a. This brings Map1 in an ideal position to act on nascent polypeptide chains as soon as they emerge from the ribosomal tunnel into the cytoplasm.

### Cryo-EM structure of the NatB-ribosome complex

**Fig 2:**
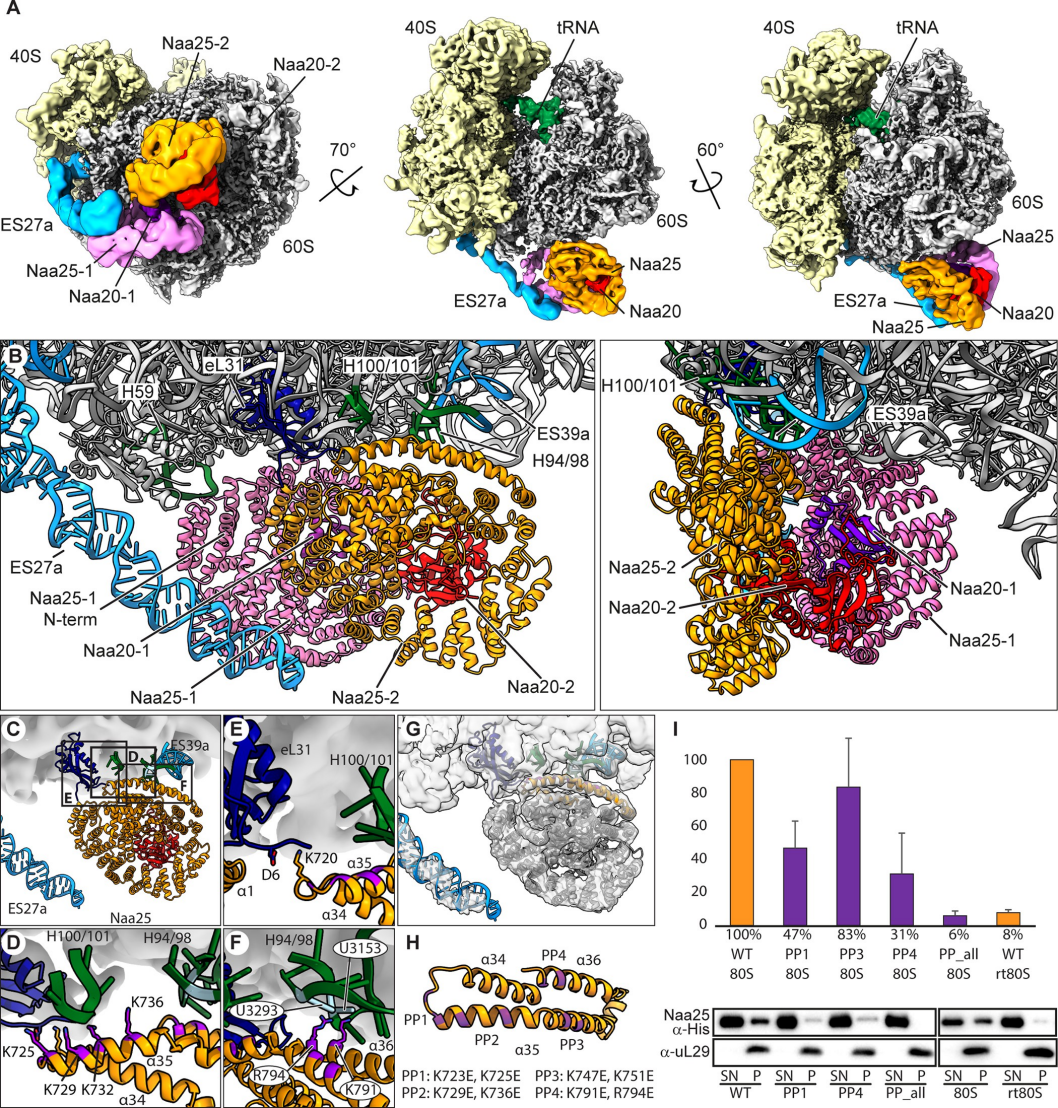
Cryo-EM structure and model of the NatB-ribosome complex. (**A**) Composite map showing cryo-EM structures of NatB in complex with translating 80S ribosomes (RNC_MDEl_) filtered according to local resolution. Isolated densities of NatB-1 (from class I) and NatB-2 (from class II) were extracted after the final refinement. Views are shown on the peptide tunnel exit (left; bottom view), rotated 70° horizontally (middle; front view), and rotated 60° vertically (right; side view). (**B**) Zoom on the peptide exit site showing the NatB-ribosome molecular model (NatB-1 and NatB-2) in front (left) and side views (right) as indicated in (**A**). Overview (upper left panel (**C**)) and zoomed views (**D**-**F**) focusing on the Naa25-2 ribosome contact sites. Interactions of (**D**) helix α35 at the C-terminus of Naa25-2 with the H100/101-and H94/98 junctions, (**E**) the Naa25-2 α34-α35 loop (Lys720) with Asp6 in the N-terminus of eL31, and (**F**) Lys791 and Arg794 at the very C-terminus (α36) of Naa25-2 with U3153 and U3293 within H94/98-junction are shown. NatB-1 was omitted for clarity. (**G**) Same view as (**C**) showing the model for the NatB-2 ribosome complex docked in density and highlighting Naa25-2 C-terminus (orange). (**H**) Model of the Naa25 C-terminus highlighting the position of the four positive patches (PPs). All patches contain two charged amino acids as indicated. Charge inversion double mutants were generated (PP1, PP3, PP4, and PPall). (**I**) Western blot analysis of sedimentation assays (triplicates) using recombinant wild-type or mutant NatB complex and idle (80S) or RNaseI-treated 80S ribosomes (rt80S). Top: fraction of NatB bound to ribosomes quantified by densitometric analysis of western blot images. Bottom: representative western blot displaying supernatant (SN) and pellet (P) fractions of two such experiments. See S1 Raw Images for all raw western blot images and S1 Data for numerical data underlying the quantification.

To gain further insight into the coordination between methionine cleavage and N-acetylation, we followed an in vitro reconstitution approach using purified components. We purified RNCs carrying a well-established NatB substrate as nascent chain, in which the free N-terminus ends with the amino acid sequence MDEL (RNC_MDEL_). The same sequence was used in form of a CoA-Ac-MDEL inhibitor for co-crystallization with *Chaetomium thermophilum* Naa20 [28]. High salt-washed RNC_MDEL_ were reconstituted with an 18× molar excess of recombinantly purified NatB (Naa25/Naa20) and subjected to cryo-EM and single particle analysis (S7 Fig). 3D variability analysis in CryoSPARC and focused sorting on the exit tunnel region revealed classes with extra density accounting for the NatB complex associated with the 80S ribosome, yet displaying a high degree of compositional and conformational heterogeneity (S8 Fig). Classes containing additional density corresponding to NatB could be divided into two sets: one set with extra density for only one copy of NatB (NatB-1; consisting of Naa20-1 and Naa25-1) flexibly attached to ES27a and one set with additional density for a second NatB complex bound to UAS-II [29] (Fig 2A). The second NatB complex (henceforth referred to as NatB-2) generally exhibited low conformational variance in classes where it was present, and its interaction with the ribosome was well resolved. To address the fact that ES27a-bound NatB-1 exhibited greater conformational heterogeneity, we performed focused sorting on this density, revealing one class (9.645 particles) in which both NatB complexes showed secondary structure resolution. Here, the ES27a-bound NatB-1 complex was positioned in direct vicinity to NatB-2 and exhibited much lower conformational flexibility than in other classes. This class (class I) was refined to a final overall resolution of 3.8 Å (local resolution ranging from approximately 4 to 9 Å for the NatB complexes; S9 Fig, left panel), which unambiguously revealed the architecture of both NatB complexes. In addition, we subjected all particles containing NatB-2 (and flexible NatB-1) to 3D variability analysis focusing on the NatB-2 area, yielding a class (class II) containing particularly well-resolved NatB-2 (45.530 particles) and refined this class to an overall resolution of 3.1 Å (local resolution ranging from below 3 to 6 Å for NatB-2; S9 Fig, right panel).

This revealed α-helical secondary structure in regions proximal to the ribosome and allowed us to unambiguously identify the disc-shaped α-helical tetratricopeptide repeat (TPR) containing Naa25 subunit for both ribosome-bound NatBs in class I (S5E–S5J Fig). We further noticed that the globular catalytic Naa20 subunits are less well resolved (when compared to Naa25), indicative of flexibility, especially in the ES27a-bound NatB-1. We then performed rigid-body fitting of an AF2 model which is highly similar to the crystal structure of *C*. *albicans* NatB (PDB 5K18) [26]. In brief, the 12 tetratrico (TPR-) repeats (α0-α29) of Naa25 together with its C-terminal helical domain (α30-α36) form a ring-like structure with N-and C-termini in close vicinity. Naa20 is located in and protrudes from a circular pocket formed by the Naa25 TPR repeats. This structure could be fitted with only minor adjustments into both NatB densities (S5E–S5J Fig and S1 Table).

Overall, the two NatB densities cover the area below the 60S exit site spanning from ES27a to the second UAS for exit factors (eL31 and uL22) [29]. NatB-1 is anchored between H59 of 25S rRNA and the long arm of the ES27a A-helix (Fig 2B). Here, contacts are established by the loops of Naa25-1 N-terminal TPRs (TPR1 and 2) that are sandwiched between the two rRNA elements. Another contact to the ES27a tip is established by the TPR-helices of the Naa25-1 C-terminus (α34-α36). In this conformation, the catalytic subunit Naa20-1 faces towards the exit tunnel, but density is only visible at the well-conserved contact interface with Naa25-1 (including highly conserved Thr2 and Glu48 of Naa20 and Arg296 of Naa25) [40], indicating that it is largely delocalized.

NatB-2 is anchored to the ribosomal surface somewhat offset from the tunnel exit of the 60S and is arranged such that the two catalytic Naa20 subunits face each other. In contrast to NatB-1, ribosomal contacts are in this case established mainly via rRNA but also to the ribosomal protein eL31, yet involving only the C-terminal TPRs of Naa25-2 (Fig 2C). In detail, we identified three distinct interaction sites of Naa25-2 with the ribosomal exit site in the map after focused refinement on NatB-2 (class II): The first site was established around the junction of H100 and H101 of 25S rRNA and the N-terminal part of α35 of Naa25-2 (Fig 2D) that contains a series of positively charged amino acids (Lys725, Lys729, Lys732, and Lys736). The second contact site is established between the Naa25-2 Lys720, located in the loop between α34 and α35, and Asp6 of the N-terminus of ribosomal protein eL31 (Fig 2E).

The third site comprises bases at the junction of H94 and H98 that bind the very C-terminus (α36) of Naa25-2 (Fig 2F). Here, the bases U3153 and U3293 within the H94/98-junction were contacted by Lys 791 and Arg794 at the very C-terminus of Naa25-2, likely via a cation-π stack.

To test the contribution of the before-mentioned residues to ribosome binding, we selected positive patches (each patch containing two closely spaced basic amino acids) at the C-terminus of Naa25 (Fig 2G) and an unrelated positive patch in the same area (Lys 747 and Lys 751). We created double charge inversion mutants (Lys or Arg to Glu) for each patch (PP1 to PP4; Fig 2H) or for all patches (PPall) similar to as described in ref [41]. Purified wild type (wt) and mutant NatB complexes carrying an N-terminal His-tag on Naa25 (for western blot detection) were used for in vitro binding assays. To prevent any bias by a specific nascent chain, we chose purified idle 80S ribosomes over RNCs in these assays (S7 Fig). The western blot analysis showed that binding of NatB to 80S ribosomes was significantly reduced by mutation of K723E, K725E (positive patch PP1; 47% of wt binding) and K791E, R794E (PP4; 31%), whereas K747E, K751E that in our structure are not directly involved in ribosome binding showed only a very weak effect (PP3; 83%). When all positive patches (PPall) were mutated, binding was almost entirely abolished (6% of wt binding), confirming the contribution of these positive patches to the interaction of NatB with the ribosome (Fig 2I). This indicated that the positive charges on the surface of the Naa25 C-terminus have an additive effect on ribosome binding by establishing a composite binding patch for rRNA interaction. The results of these binding assays confirm the significance of the described interaction patches between Naa25 and the ribosome. Whereas in Naa25-1, these positively charged amino acids interact with ES27a, in Naa25-2, they enable the binding to H94/H98 and H100/H101 junctions (Fig 2D–2F).

Interestingly, as observed for NatA and also Map1, in the class showing the stable assembly with two NatB complexes (class I), ES27a adopts a specific conformation. Compared to the Map1-C1 position of ES27a that is closest to the tunnel exit, the NatB-bound position is rotated 37° away from the tunnel exit (S6 Fig).

We thus assessed the contribution of ESs to NatB binding by performing quantitative binding assays using empty 80S or RNAse-I-treated 80S (depleted of ES as described in [25] and for Map1 in Fig 1I) (S7 Fig). The absence of ESs indeed reduced the binding of NatB to 8% compared to NatB binding to untreated 80S ribosomes, confirming an important role of the ES for recruitment of both NatB copies to the ribosome (Fig 2I).

**Fig 3:**
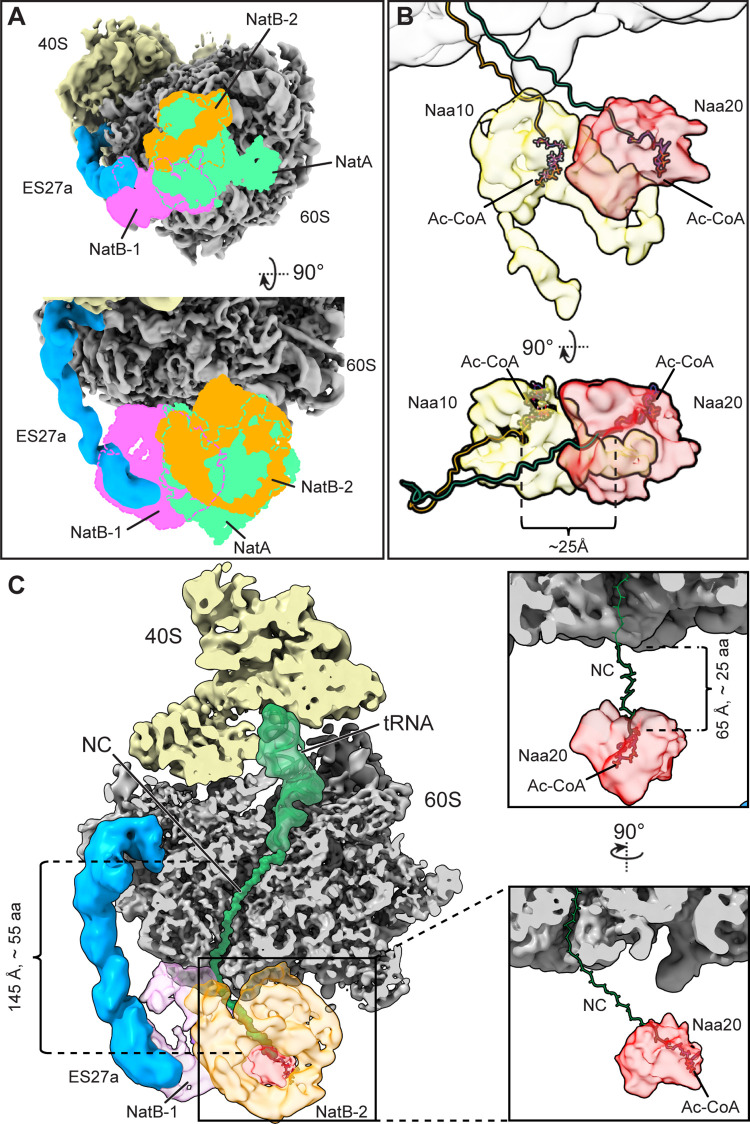
Comparison of ribosome-bound NatB and NatA complexes. (**A**) Bottom view (upper panel) and front view (lower panel) showing an overlay of the NatB-1 (pink) and NatB-2 (orange) ribosome structure with isolated densities for ribosome-bound NatA (bright green) (EMD-0201; [25]). (**B**) Comparison of positions for the NatA and NatB-2 catalytic subunits (Naa10 and Naa20) with respect to the 60S subunit shown as front and top view. The position of acetyl-CoA (Ac-CoA) and a putative model for the nascent chains is shown. For clarity, only Naa20 of NatB-2 is shown. (**C**) Left panel: cut front view of the NatB ribosome cryo-EM map highlighting the nascent polypeptide chain and the position NatB-1 and NatB-2 (left panel). Right panels: Zoom-in views highlighting the catalytic Naa20-2 subunit and illustration of the minimal distance that a nascent chain has to span to reach the active site of Naa20-2.

We next compared ribosome-bound NatB-1 and NatB-2 with the NatA complex. Here, several observations were made. (i) The overall space occupied below the exit site is overlapping, indicating that in the observed conformations NatA and NatB can only bind exclusively (Fig 3A). (ii) The architecture of ribosome-bound NatB clearly differs from the NatA-ribosome complex and displays a distinct 60S binding mode. NatA mainly employs 25S rRNA ES for 60S binding. Here, ES27a and ES39 anchor the auxiliary Naa15 (Nat1) subunit to the exit site, and Naa50 (Nat5)—which has no equivalent in NatB—makes a third contact to ES7. While NatB-1 also binds to ES27a, NatB-2 engagement of the 60S differs compared to NatA binding and does not involve ES27a or other ESs. (iii) Like Naa10 (Ard1), the catalytic subunit of the NatA complex, both Naa20-1 and Naa20-2 of NatB have no direct contact to the ribosome. We further note that Naa20-2 is better resolved, while Naa20-1, apart from the contact site with Naa25-1, is largely delocalized. Notably, a rigid body fit of the NatB-2 model into NatB-1 would lead to a clash between Naa20-1 and Naa25-2, indicating that Naa20-1 needs to adjust its orientation with respect to Naa25 compared to NatB-2 (and the X-ray structure [26]). Nevertheless, in order to assess their principal potential to contribute catalytic activity, we compared this rigid-body fit of Naa20-1 with our models for Nat20-2 and Naa10 of NatA, since it represents a sufficiently accurate approximation of the overall position of Naa20-1 (Figs 3C and S10).

Interestingly, it would require roughly the same minimum length of the nascent chain of about 55 amino acids to reach either one of the catalytic centers, assuming a direct path from the 3′-CCA end of the tRNA to the tunnel exit and from there into the Naa20 catalytic center (30 aa inside and 25 aa outside the exit tunnel) (Fig 3C). Naa20-2 is oriented similarly to Naa10 of NatA [25] with respect to the position of acetyl-CoA and accessibility for the nascent chain N-terminus (Fig 3B and 3C). While the substrate could enter Naa20-2 in a straight path from the tunnel exit, it would need to form a turn to reach into the center of Naa20-1, the entrance to which is located on the lateral side (S10 Fig). Thus, both copies of Naa20 (in NatB-1 and in NatB-2) could in principle be catalytically active. Yet, taking into account the delocalization and high degree of flexibility of Naa20-1 with respect to its auxiliary subunit in contrast to the more stably positioned Naa20-2, and given the more direct path that the nascent chain can take to enter Naa20-2, we speculate that Naa20-2 rather than Naa20-1 would act to N-acetylate most NatB substrates.

While binding of NatA and NatB appears mutually exclusive we wondered to what extent concomitant binding of Map1 would be sterically allowed. Unlike stated before [25], comparison of the binding modes reveals that Map1 in both C1 and C2 positions would possibly clash with NatA, although clashes between Map1-C1 and NatA would be rather minor. Yet, both NatB complexes, especially the ES27a-bound NatB-1, would severely overlap with both observed Map1 positions (S11 Fig). Thus, this comparison is rather suggestive for competitive binding of Map1 and NATs. This notion is further supported by the observation that ES27a orientations are apparently different for every ligand.

## Discussion

During translation of a nascent polypeptide, various factors are dynamically interacting with the ribosomal peptide exit site to probe the biochemical and biophysical properties of the emerging nascent chain. For example, the activity of the modifying enzymes Map1 and the NATs is dependent on the properties of the amino acids following the N-terminal methionine. The ribosome-associated complex RAC containing the Hsp70 homolog Ssz1 binds various (partially unfolded) nascent chains, whereas the SRP recognizes hydrophobic, partially helical N-terminal signal sequences.

**Fig 4:**
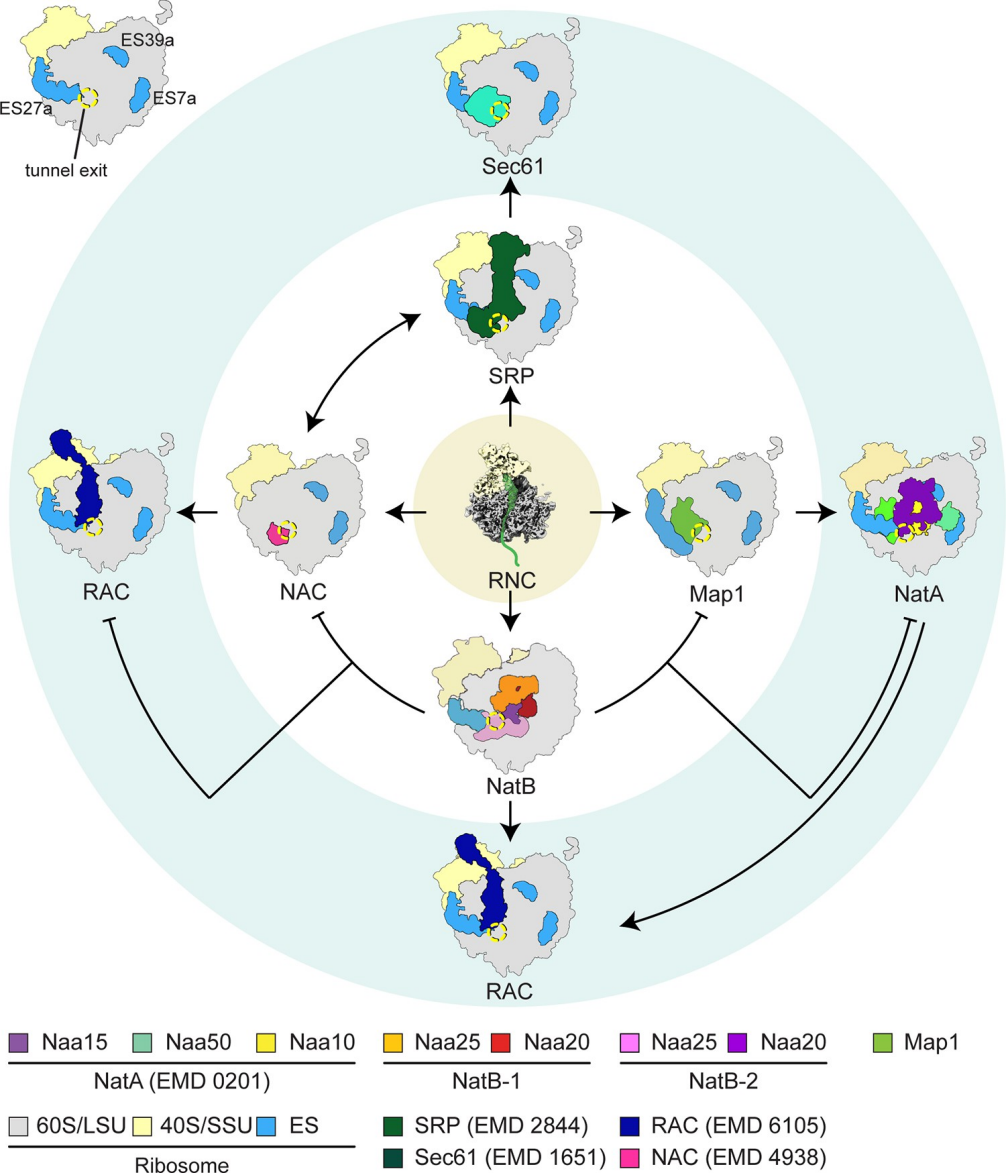
Scheme depicting the possible interplay of ribosome exit site factors. The translating ribosome exposing a nascent polypeptide chain is engaged by primary exit site factors (NAC, Map1, NatB, SRP) depending on the properties of the emerging chain’s N-terminus. After these primary factors have performed their activity, secondary factors (e.g., NatA, RAC, Sec61) gain access to the nascent chain. Whereas some of them can coexist on the ribosome (e.g., NatA and Map1-C1 or NAC and SRP), others cannot or have to bind sequentially (e.g., NatA and NatB). ES: expansion segment, RNC: ribosome-nascent chain complex. The tunnel exit is indicated by a yellow dotted circle. Color codes for factors and entry codes for the electron microscopy database (EMD) are given below. Reference for EMD 2844 is [45], for EMD 6105 is [46], for EMB 1651 is [47], and EMD 4938 is [48].

Commonly, all these factors are able to interact with the ribosome employing fast on- and off-rates even in the absence of the nascent chain in order to scan the ribosome for the emerging nascent chain substrate. Yet, a number of structural studies showed that binding sites for most exit site factors on the ribosome are overlapping. Based on those structures, neither NatA nor RAC or SRP can bind together to the exit site, at least not in the observed conformations. This implies a dynamic and sequential or collaborative ribosome interaction and activity of these factors on the nascent chain dependent on the presence of the cognate nascent chain N-terminus substrate, which will change the apparent off-rates of the respective factors (see scheme in Fig 4). This is best documented for SRP, which, after an initial encounter, remains bound to the RNC for the entire targeting cycle, but only after engaging a sufficiently hydrophobic signal sequence [42–44].

This study investigating ribosome binding for Map1 and NatB expands our knowledge on how exit site factors on the ribosome may be orchestrated by the dynamic rRNA expansion segment ES27a. We noted that, as observed for the NatA complex, Map1 and NatB directly bind to ES27a and intriguingly position the rRNA A-helix in a very specific conformation with respect to the tunnel exit (see S6 Fig). Map1, which cleaves the N-terminal methionine, is likely one of the earliest nascent chain binders, since cleavage at least for bacterial MAP has been shown to occur as soon as the nascent chain has reached a length of 44 amino acids [49]. We find it mainly bound to ES27a and were able to enrich two distinct conformations positioning the Map1-bound tip of ES27a closer to the tunnel exit than observed with NatA. In contrast, when bound to the two NatB complexes, ES27a moves even further away from the tunnel exit than in case of NatA binding. We thus hypothesize that ES27a, by adopting exit factor-specific conformations, plays a role in providing specificity and probably exclusive binding for the different factors.

Apart from the ES27a interaction, most exit site factors employ electrostatic interactions to bind to 25S/28S rRNA. For example, positively charged patches are present in NAC [29,48,50], NatA, bacterial Map [49], and the RAC/Ssb1 complex [46,51,52]. Interestingly, the precise electrostatic interaction sites on the ribosome differ from factor to factor. Both NatA and NatB employ charged patches on their TPR-repeat containing subunits. Naa15 of NatA contacts a binding pocket formed by rRNA H24 and H47 close to eL31 with its charged N-terminus, and the region close to ES39 (H98-H100 region) with a patch of lysines on its C-terminus. As described above, both Naa25 subunits in our structure employ positively charged patches to engage with rRNA, whereby Naa25-2 binds to a similar but not the same region of the exit site (see Fig 2C–2F) as Naa15 (NatA), thus showing a different specific binding pattern.

We propose a model according to which the exit site factors Map1, NatA, and NatB all require an interaction with the flexible arm of ES27a. In addition, however, binding of these factors to the ribosomal exit site specifically positions both the factors themselves and also ES27a in distinct states, thereby enabling probing of the nature of the nascent chain as potential substrates.

We thus speculate that, in analogy to the mobile P-stalk for translation factors [53–55], ES27a could be a primary binding hub for modifying enzymes, maintaining them in close vicinity to the nascent chain substrates for probing their properties. More rigid ribosome binding can then occur via factor-specific interactions mainly involving electrostatic interactions with the exit site rRNA, leading to both specific fixation of ES27a and temporary exclusion of other competing exit site factors.

Substrates of NatA-mediated N-acetylation require prior removal of the first methionine by Map1, whereas for NatB activity, the N-terminal methionine still needs to be present. Interestingly, in our structures, we observe two copies of NatB, one rigidly bound next to the peptide exit tunnel (at UAS-II) with no contact to ES27a, and one more flexibly bound mainly to ES27a. We further show that presence of ES27a is necessary for both NatBs to bind to ribosomes (Fig 2I), since removal of ES27a by RNaseI almost completely abolishes ribosome binding. This suggests that spatial constraining and correct positioning of NatB-2 (which does not contact ES27a) is likely to depend on the presence of NatB-1 and ES27a. This is further supported by the observation that, during classification, all classes that showed NatB-2 also contained extra density for NatB-1 at ES27a, whereas not all classes with NatB-1 showed NatB-2. This opens the possibility that NatB-1 binds first to the ribosome via ES27a and is needed to stably position NatB-2 next to the tunnel exit. This would be analogous to a recent study showing that exit factors such as NAC and SRP can in principle cooperate [56]. For cotranslational ER-targeting, NAC acts as a gatekeeper to shield nascent chains, which are not substrates for SRP, while facilitating recruitment of SRP to the ribosome. Another study shows that the Translocon Associated Protein (TRAP) may help recruiting ribosomes to the ER and subsequently aids in stabilizing the RNC-Sec61 complex and contributes to membrane protein biogenesis [57].

In contrast to NatB, concomitant binding of NatA and Map1-C1, but not Map1-C2, is still possible since they would barely sterically clash (S11 Fig). Interestingly, bacterial Map also occupies two different positions on the ribosomal surface close to the exit tunnel, only one of which allows binding of peptidyl-deformylase (PDF) at the same time [36]. Given that yeast Map1 is bound to the ribosome mainly via ES27a and its position is dependent on ES27a movement, we favor a model for a Map1-NatA interplay, in which ES27a orchestrates a sequential action of Map1 and NatA on their substrate. This is in line with a failure of all our attempts to visualize Map1 and NatA together on the ribosome.

A possible reason for the somewhat puzzling observation of two NatB copies on the ribosome might reflect a function in efficient discrimination between Map1, NatA, and NatB substrates. Among the cotranslationally acting nascent chain modifying factors discussed in this study, Map1 is the most abundant protein (average copy number per cell according to the *Saccharomyces* Genome Database (www.yeastgenome.org; 14,218 +/− 5,474) followed by NatA (Naa15; 8,398 +/− 4,076) and NatB (Naa25; 5,693 +/−1,503). Thus, the likelihood for an RNC to be probed by Map1 or NatA is higher. If, however, the first NatB binds to RNCs with a vacant ES27a, it would automatically exclude Map1 or NatA (re) binding and thereby prime this RNC as a possible substrate for the second, likely the catalytically active, NatB-2. At this point, however, we cannot clearly decide whether both or only one of the NatB copies are active in modifying nascent chains. Taking into account the observed conformational distortion of Naa20 of NatB-1 and the rather obscured path of the nascent chain to its catalytic subunit, we speculate that NatB-2 may be the more active complex that provides the majority of the modifying activity.

Taken together, we propose that the primary and secondary interactors of the nascent chain (see Fig 4) may follow either a collaborative (as in case of NAC and SRP) or a sequential mode (as in case of Map1 and NatA) for the productive interplay of the various modifying enzymes. At least for the nascent chain modifying enzymes, ES27a plays a central role in their recruitment to and orchestration at the ribosomal peptide exit site. However, for a complete understanding, a quantitative assessment of the kinetic properties of the different modifying, chaperoning, and targeting factors with respect to nascent chain-dependent RNC binding and dissociation will be necessary.

## Materials and methods

### Purification of recombinant Map1

Map1 containing an N-terminal His_8_-tag followed by a linker and a HRV 3C cleavage site was expressed from a pET28a vector. *E*. *coli* cells were harvested by centrifugation in a SCL6000 rotor (Sorvall) for 10 min at 4,500 rpm and 4°C. After washing with 1× PBS, the pellet was resuspended in cold lysis buffer (50 mM Tris (pH 8.0), 500 mM NaCl, 1 mM PMSF, 1 EDTA-free protease inhibitor cocktail pill/50 ml (Roche)) and subjected to mechanical lysis using a Microfluidizer (Micro Fluidics). Clarified lysate was obtained after spinning for 15 min at 15,000 rpm in a SS34 rotor (Sorvall) at 4°C. After filtering the lysate, it was applied on a HisTrap HP column (GE Healthcare) equilibrated with HT-20 buffer (50 mM Tris (pH 7.5), 500 mM NaCl, 20 mM imidazol, 10 mM β-mercaptoethanol). After elution with HRV 3C protease under high salt conditions, the main fractions were loaded onto a SP FF column (GE Healthcare) equilibrated with 20 mM HEPES at pH 7.0. Elution was performed by a linear salt gradient up to 1 M NaCl, the main fractions showing purified Map1 protein were pooled and the buffer was adjusted to 20 mM HEPES (pH 7.5), 100 mM KOAc, 2.5 mM Mg(OAc)2, 1 mM DTT, 0.5 mM PMSF, 10 μg/ml cycloheximide including a protease inhibitor cocktail tablet (Roche).

### Purification of recombinant NatB complexes

Naa25 containing an N-terminal His_8_-tag followed by a linker and a HRV 3C cleavage site was expressed from the MCS1 of pRSFDuet-1 vector (Novagen). A gene encoding a codon-optimized version of catalytically inactive E25A and H74A double mutant of Naa20 [26] for *E*. *coli* expression was synthesized by Eurofins. An additional M36L mutation was introduced to prevent internal translation initiation. This modified *NAA20* gene was cloned into the MCS2 of the same pRSFDuet-1 vector. To overcome a disproportion of auxiliary to catalytic subunit, Naa20 was also cloned into pET21a separately and was used for coexpression with the dimeric NatB construct. After transformation of both plasmids into *E*. *coli* BL21(DE3) cells, cultures were grown in LB medium and induction was performed with 1 mM IPTG at 16°C overnight. Harvesting and lysate preparation was done as described for Map1.

NatB and its mutants (PP1 to PPall) were purified via Ni-NTA following the manufacturer’s protocol with the exception that after washing proteins were eluted in 50 mM MES (pH 6.0), 500 mM NaCl, and 500 mM imidazole. Eluted NatB was subsequently subjected to size exclusion chromatography on a Superdex 200 (GE Healthcare) in GF buffer (10 mM MES (pH 6.0), 400 mM KOAc, 5 mM Mg(OAc)_2_, and 1 mM DTT). NatB containing fractions were pooled and concentrated using Ultra-4 centrifugal filter devices (Amicon, MWCO 50 kDa) and stored in GF buffer at −80°C.

For the cryo-EM sample, the His-tag was removed by 3C protease. About 200 μg of His_8_-NatB in GF buffer were incubated with 25 μg His_6_-3C protease for 45 min at 20°C on a turning wheel. Cleaved His_8_-tag and His_6_-3C protease were removed using 10 μl of magnetic beads (His-tag Isolation & Pulldown; Thermo Fisher), and the supernatant was used for reconstitution of the NatB-RNC_MDEL_ complex for single particle analysis.

### Generation and purification of charge inversion mutant NatB complexes

Several positive patches on Naa25 were identified as potential candidates for ribosome binding: patch1 containing K723 and K725, patch2 containing K729 and K736, patch3 containing K747 and K751, and patch4 containing K791 and R794. We generated charge inversion mutants of all four positive patches (PP1 to PP4) mutated to E (similar to Magin and colleagues [41]), as well as one mutant with all PP amino acids mutated to E. Mutations were performed using a site-directed mutagenesis kit (New England BioLabs). Mutation in the DNA sequence were introduced by PCR. To that end, the pRSFDuet-1 vector harboring the NAA25 insert (see above) was amplified using primers introducing charge inversions. Site-directed mutagenesis was performed according to the manufacturer’s manual. Expression and purification were performed in the same way as described above.

### Purification of native Map1-ribosome complexes

For native pullouts of Map1-ribosome complexes, a *S*. *cerevisiae* strain expressing C-terminally tandem affinity purification (TAP)-tagged Map1 from Euroscarf (genotype SC0000; MATa; ura3-52; leu2-3,112; YLR244c::TAP-KlURA3; accession number SC1694) was used. Cells were grown in YPD medium to an OD_600_ of 4.0 and 5 g of wet cells were resuspended in lysis buffer (LB-2.5; 20 mM HEPES (pH 7.5), 100 mM KOAc, 2.5 mM Mg(OAc)_2_, 1 mM DTT, 0.5 mM PMSF, 10 μg/ml cycloheximide, protease inhibitor cocktail tablet (Roche)). Cell disruption was performed using a Freezer Mill (6970 EFM). The powder was resuspended in 15 ml LB-2.5 and the lysate spun for 15 min at 4°C in an SS-34 rotor (Sorvall) at 15,000 rpm to clarify the lysate. The SN was loaded onto several 600 μl sucrose cushions (750 mM sucrose in LB-2.5) and centrifuged for 1 h at 100,000 rpm in a TLA 100.3 (Sorvall) at 4°C. The pellets were resuspended in LB-2.5 and pooled for subsequent TAP purification. Approximately 150 μl of magnetic IgG-coupled Dynabeads M-270 Epoxy (Life Technologies) were equilibrated with 300 μl LB-2.5 containing 0.5% TritonX-100 (LB-2.5+T) twice and added to the pooled sample. The sample was incubated with the beads for 1 h at 4°C on a rotating wheel, harvested on a magnet, and resuspended in 500 μl LB-2.5+T. After three washing steps with 500 μl LB-2.5 and three washing steps with 500 μl LB-2.5 (sample: W4-6), ribosome-Map1 complexes were eluted in 120 μl LB-2.5 containing 70 units of Ac-TEV protease (Thermo Fisher) for 1 h at 20°C. A concentration of 14 A_260_/ml was measured on the NanoDrop (Implen). Samples were subsequently analyzed on a 12% Nu-PAGE gel followed by western blot analysis.

### Mass spectrometry of native Map1-ribosome complexes

Proteins were in-gel reduced for 30 min at 55°C using 45 mM dithioerythritol (DTE) in 50 mM NH_4_HCO_3_. Cysteines were carbamidomethylated for 30 min at room temperature in 100 mM iodoacetamide/50 mM NH_4_HCO_3_. In-gel digestion was performed at 37°C overnight with 70 ng porcine trypsin (Promega, Fitchburg, WI, USA). Peptides were extracted using 70% ACN. Prior to liquid chromatography, the samples were dried using a SpeedVac vacuum concentrator. Peptide chromatography was performed on an Ultimate 3000 nano-LC system (Thermo Fisher Scientific) using an EasySpray separation column (PepMap RSLC C18, 50 cm length, 75 μm ID, Thermo Fisher Scientific) at a flow rate of 250 nl/min. As solvent A 0.1% formic acid was used. The chromatography method consisted on gradients from 3% to 25% solvent B (0.1% formic acid in acetonitrile) in 30 min and from 25% to 40% B in 5 min. Mass spectrometry was performed on a Q Exactive HF-X mass spectrometer (Thermo Fisher Scientific) using a top 12 data-dependent acquisition method. Spectra were searched using MASCOT V2.4 (Matrix Science, London, UK) and the *S*. *cerevisiae* subset of the UniProt database. The results were filtered for an FDR <1%.

### In vitro translation and purification of ribosome nascent chain complex

Ribosome nascent chain complexes were purified after in vitro translation of an mRNA reporter in a cell-free yeast translation extract. For reconstitution with Map1, the previously described truncated uL4 construct [25] containing an N-terminal His_8_-HA tag for purification and immunoblotting followed by a TEV cleavage site, the first 64 amino acids of uL4 and a “CMV” stalling sequence was used (His-HA-TEV-CMV-uL4 mRNA). For reconstitution with NatB, the abovementioned construct was modified to code for a NatB substrate. The first five residues of uL4 were replaced by a MDEL sequence, which is preceded by a His_8_-V5 tag followed by a Factor Xa cleavage site.

His-V5-Xa-CMV-uL4 mRNA and His-HA-TEV-CMV-uL4 mRNA was produced using the T7 Message Machine Kit (Thermo Fisher). For preparation of uL4-CMV-RNCs or uL4-CMV-RNC_MDEL_, ribosomes were programmed using a yeast cell-free translation extract, either from *ski2*Δ cells (for uL4-RNC) or BY4741 cells (for uL4-RNC_MDEL_). The in vitro translations were performed at 17°C for 75 min as described before and stopped by adding 200 μg/ml cycloheximide (only for uL4-CMV-RNCs). The uL4-CMV-RNCs were affinity purified using magnetic Ni-NTA beads (Dynabeads). For this, the translation reaction was mixed with preequilibrated Dynabeads in 250 buffer (50 mM Tris/HCl (pH 7.0), 250 mM KOAc, 25 mM Mg(OAc)_2_, 5 mM β-mercaptoethanol, 250 mM sucrose, 10 μg/ml cycloheximide, 0.1% Nikkol, 0.1% EDTA-free protease inhibitor cocktail pill (Roche), 0.1% SUPERase-In, 20 U/l (Thermo Fisher)) containing 10 μg ml^−1^ yeast tRNA mix (Sigma-Aldrich) for 15 min at 4°C, using a 800 μl slurry of beads for a 1,250-μl sample. The bead resin was washed 3 to 4 times with 250 buffer. Elution was performed using 250 buffer with 350 mM imidazole over the course of 5 min. The sample was loaded onto 400 μl of high salt sucrose cushion (1 M sucrose 50 mM Tris/HCl (pH 7.0), 500 mM KOAc, 25 mM Mg(OAc)_2_, 5 mM β-mercaptoethanol, 10 μg/ml cycloheximid, 0.1% Nikkol, 0.1% EDTA-free protease inhibitor cocktail pill (Roche)), and ribosomes were pelleted by centrifugation using a TLA 120.2 rotor (Beckman) for 45 min at 100,000 rpm and 4°C and resuspended in 30 μl 250 buffer on ice for 30 min while shaking. Subsequently, the N-terminal His-HA-tags were cleaved using TEV protease in 250 buffer for 45 min at room temperature. The mixture was again spun through a 600-μl sucrose cushion in a TLA 100 rotor (Beckman) for 45 min at 100,000 rpm and 4°C. Afterwards, the pellet was resuspended in 30 μl grid buffer (20 mM Tris/HCl (pH 7.0), 50 mM KOAc, 2.5 mM Mg(OAc)_2_, 1 mM DTT, 125 mM Sucrose, 100 μg/mL cycloheximide, 0.05% Nikkol) on ice while shaking for 30 to 45 min.

uL4-CMV-RNC_MDEL_ were purified as described above with following modifications: In all buffers, 50 mM HEPES/KOAc (pH 7.5) was used instead of Tris/HCl (pH 7.0), and cycloheximide was omitted from all buffers. Before elution, an additional high-salt wash was added (with 50 mM HEPES/KOAc (pH 7.5), 500 mM KOAc, 25 mM Mg(OAc)_2_, 5 mM β-mercaptoethanol, 250 mM sucrose, 0.1% Nikkol, 0.1% EDTA-free protease inhibitor cocktail pill (Roche)). After centrifugation, the ribosomal pellet was resuspended in Factor Xa cleavage buffer (20 mM HEPES/KOH, 150 mM KOAc, 5 mM Mg(OAc)_2_, 5 mM Ca(Cl)_2_ 125 mM Sucrose, 5 mM β-mercaptoethanol, 0.1% Nikkol). To cleave the His_8_-V5 tag and to obtain the free MDEL N-terminus, Factor Xa protease was added to resuspended RNCs to a final concentration of 0.25 mg/ml, and the sample was incubated for 3 h at room temperature on a rotating wheel. Subsequently, the reactions were spun again through the high salt sucrose cushion (see above) uL4-CMV-RNC_MDEL_ (in short RNC_MDEL_), and pellets we resuspended in grid buffer (20 mM HEPES/KOH, 100 mM KOAc, 5 mM Mg(OAc)_2_, 125 mM sucrose, 5 mM β-mercaptoethanol, 0.05% Nikkol).

### Purification of nonprogrammed 80S ribosomes

Idle, nonprogrammed 80S ribosomes were purified from *S*. *cerevisiae* W303-1a cells. Cells were harvested at logarithmic growth, resuspended in lysis buffer (20 mM HEPES-KOH (pH 7.5), 100 mM KOAc, 10 mM Mg(OAc)_2_, 1 mM DTT, protease inhibitor (Roche)), and opened up using a French press. Cell debris was separated by centrifugation (SS34 rotor, 15,000 rpm, 20 min at 4°C). The supernatant was cleared by another centrifugation (Ti70 rotor, 37,000 rpm, 30 min at 4°C) resulting in an “S100 extract.” About 3 ml of this S100 supernatant were loaded on 1 ml sucrose cushion (20 mM HEPES-KOH (pH 7.5), 500 mM KOAc, 10 mM Mg(OAc)_2_, 1 mM DTT, protease inhibitor (Roche), and 1.5 M sucrose), and ribosomes were pelleted (TLA110 rotor, 100,000 rpm, 1 h, 4°C). The pellet was resuspended in 300 μl Buffer A (20 mM HEPES-KOH (pH 7.5), 500 mM KOAc, 12.5 mM Mg(OAc)_2_, 1 mM DTT), mixed with an equal volume of 2× puromycin buffer (20 mM HEPES-KOH (pH 7.5), 500 mM KOAc, 12.5 mM Mg(OAc)_2_, 1 mM DTT, 2 mM puromycin, and 0.01% RNasin), and incubated for 30 min at 25°C. The reaction was then loaded on a 10% to 40% sucrose gradient in buffer A and spun for 20 h (SW40 rotor, 13,400 rpm, 4°C). The gradient was harvested using a gradient station (Biocomp), the 80S peak was collected, the ribosomes were concentrated by pelleting (TLA110, 100,000 rpm, 1 h, 4°C), and the 80S ribosomes were resuspended in lysis buffer.

### RNase I treatment of 80S ribosomes and ribosome nascent chain complexes

For selective removal of eukaryotic specific expansion segments, RNCs or nonprogrammed ribosomes (80S) were incubated with 40 U RNase I (Thermo Fisher) per 1 A_260_ unit of ribosomes for 45 min at 25°C. The reaction was stopped with 0.5 U SUPERase-In (Thermo Fisher) RNase inhibitor per 1 U of RNase I, and the sample was placed on ice. The RNase I-treated 80S and RNCs are referred to as rt80S and rtRNC, respectively.

### In vitro reconstitution of Map1-ribosome complexes

To characterize binding of Map1 to ribosomes, Map1 was in vitro reconstituted with uL4-RNC or RNaseI-treated uL4-RNC (rtRNC). Binding reactions were performed using 2 pmol of ribosomes (80 nM) and a 30-fold molar excess (2,4 μM) of purified Map1. The binding buffer was adjusted to a final concentration of 50 mM Tris (pH 7.0), 150 mM KOAc, and 2.5 mM Mg(OAc)_2_. After preincubation of all components but ribosomes for 5 min on ice, the 2 pmol ribosomes were added and the samples were incubated for 15 min at room temperature. The reaction was loaded onto 600 μl sucrose cushion (750 mM sucrose, 20 mM HEPES (pH 7.5), 150 mM KOAc, 2.5 mM Mg(OAc)_2_, 1 mM DTT) and spun for 2.5 h in a SW55Ti rotor (Beckman) at 40,000 rpm and 4°C. The tubes were immediately frozen in liquid nitrogen and cut at 1/3 from the bottom. The upper 2/3 contained the supernatant (SN), whereas the lower 1/3 contained the pellet fraction (P). After TCA precipitation, Map1 binding to ribosomes was analyzed by SDS-PAGE after staining with SimplyBlue SafeStain (Thermo Fisher), and densitometry was performed using ImageJ version 1.53Q. See S1 Raw Images for all raw gel images and S1 Data for numerical data underlying the densitometric quantification.

### In vitro reconstitution of NatB-ribosome complexes

Binding assays were performed using 80S and purified NatB complex (either with wt Naa25 or PP mutant Naa25 variants). All assays were performed using 2 pmol (80 nM) of ribosomes and 20-fold molar excess of NatB (1.6 μM). The binding buffer for NatB-ribosome complex formation was adjusted to 50 mM Tris (pH 7.5), 150 mM KOAc, 5 mM Mg(OAc)_2_, and 1 mM acetyl-CoA. Sample reactions were pelleted through a sucrose cushion (750 mM sucrose, 20 mM HEPES (pH 7.5), 150 mM KOAc, 5 mM Mg(OAc)_2_, 1 mM DTT, and 10 μg/ml cycloheximide). Supernatant and pellet fractions were separated on Nu-PAGE gels and transferred onto PVDF membranes. After transfer, the lower half of the membrane was decorated with a polyclonal antiserum against uL29 and the upper half was analyzed using an anti-His antibody (Roche). Secondary antibodies were conjugated with horseradish peroxidase. Western blots were analyzed via chemiluminescence on an Amersham LAS Imager 600, and densitometry was performed using ImageJ version 1.53Q. See S1 Raw Images for all raw western blot images and S1 Data for numerical data underlying the densitometric quantification.

### Cryo-electron microscopy of the Map1-ribosome complex

#### Sample preparation

Approximately 4 pmol of the freshly prepared samples from the native Map1-TAP pullout were adjusted to 40 μl total sample volume with LB-2.5 containing Nikkol to a final concentration of 0.05% (w/v). For cross-linking, glutaraldehyde was added to a final concentration of 0.02% (w/v), and the sample was incubated for 15 min on ice. After a short spin of 5 min at 4°C and 14,000 rpm in a table top centrifuge (Eppendorf), 3.5 μl were applied to 2 nm precoated Quantifoil R3/3 holey carbon support grids. Before sample application, the grids were glow discharged for 30 s at 0.3 mbar.

#### Vitrification and data processing

Vitrification was performed by plunge freezing the grid into liquid ethane using Vitrobot Mark IV (FEI Company/Thermo Fisher) with an incubation time of 45 s and blotting for 2 to 3 s at 4°C and a humidity of 95%. Data were collected on a Titan Krios G3 (Thermo Fisher) equipped with a K2 direct detector (Gatan) at 300 keV using the semiautomated data acquisition software EPU (Thermo Fisher). A total of 48 frames with a dose of 1.17 ^e−^/Å^2^ per frame were collected in a defocus range of −0.5 to −3.2 μm. Magnification settings resulted in a pixel size of 1.059 Å/pixel. Frame alignment was executed with MotionCor2 [58], and the estimation of the contrast transfer function (CTF) was performed with Gctf [59].

Micrographs were screened manually for ice quality, and the resulting 8,358 micrographs were used for automated particle picking in Gautomatch (https://www2.mrc-lmb.cam.ac.uk/download/gautomatch-056/). After a two-dimensional (2D) classification in RELION 3.0 [60] to discard nonribosomal particles, in total, 115,082 particles were subjected to an initial refinement. Subsequent 3D classification into eight classes led to three classes only containing poorly resolved 80S ribosomes (7% with 8,234 particles, 1% with 1,226 particles, 0.01% with 149 particles) and one class showing the 60S subunit only (6%, 6,728 particles). The remaining four classes showed well-resolved 80S ribosomes, all of which were programmed with tRNAs (mainly in A/A and P/P states) and showed the mobile expansion segment ES27a solely in the exit position and a weak extra density between ES27a and the tunnel exit. One of these classes (12%, 13,804 particles) contained eRF1 and ABCE1/Rli1. These four classes were merged for further processing (86%, 98,745 particles). A subsequent 3D classification using a binary soft mask enclosing ES27a and the region below the TE revealed one class (25%, 25,245 particles) that showed a well-shaped ES27a as well as a defined extra density where Map1 was expected. All other classes showed either poorly resolved ES27a or fragmented densities at the expected Map1 position (24% with 23,499 particles, 28% with 27,590 particles, 23% with 22,411 particles). The well-resolved class was further refined and subjected to another round of local 3D classification, this time with a mask covering only the tip of ES27a and the putative Map1 density. The two resulting classes exhibited Map1 in two different conformations, either tightly connected to ES27a (47%, 11,938 particles) or with a looser connection to ES27a (53%, 13,307 particles). Both classes were CTF refined to an overall resolution of 3.9 Å and 3.8 Å according to the gold standard resolution criterion (FSC = 0.143) and comprising 10% or 12% of the total particle number after 2D classification, respectively. Subsequently local resolution was calculated using RELION.

#### Model of the Map1-ribosome complex

To interpret the density for Map1, a homology model was generated by AF2 [37].

To position this model inside the low-resolution density, it was superimposed on the high-resolution cryo-EM model of human Ebp1 bound to the ribosome (PDB 6SXO; [35]). This was followed by a rigid-body fit of the Map1 model into the two isolated densities.

### Cryo-electron microscopy of the NatB-ribosome complex

#### Sample preparation

Around 2.5 pmol uL4-RNC_MDEL_ were in vitro reconstituted with an 18-times molar excess (44 pmol) of recombinantly purified inactive NatB complex. Final buffer conditions were 50 mM HEPES (pH 7.5), 116 mM KOAc, 5 mM Mg(OAc)_2_, 1 mM DTT, 0.05% Nikkol (w/v), 1 mM acetyl-CoA. The total reaction volume for cryo-grid making was 25 μl.

#### Vitrification and data processing

The freshly prepared sample was applied to 2 nm precoated Quantifoil R3/3 holey carbon support grids and plunge frozen under the same condition as described for the Map1-ribosome complex. Data were collected on a Titan Krios G3 (Thermo Fisher) equipped with a K2 direct detector (Gatan) at 300 keV using the semiautomated data acquisition software EPU (Thermo Fisher). A total of 40 frames with a dose of 1.409 ^e−^/Å^2^ per frame were collected in a defocus range of 0.5 to 3.5 μm. Magnification settings resulted in a nominal pixel size of 1.049 Å/pixel. Frame alignment was performed with MotionCor2 [58].

All further processing steps were carried out in CryoSPARC, version 4.0.0 [61] unless otherwise specified. For a total of 10,380 selected micrographs, CTF estimation was performed using the patch-based CTF estimator in CryoSPARC. 80S ribosomal particles were picked by first generating templates from a subset of micrographs using CryoSPARC’s Blob Picker and performing 2D classification, then picking from all micrographs using the Template Picker with the thusly generated 2D templates.

After 2D classification, 447,470 particles were selected for ab initio reconstruction and homogenous refinement of a consensus map. The aligned particles were then subjected to 3D variability analysis using a soft mask around the peptide tunnel exit region on the large ribosomal subunit.

Roughly half of all particles (45.5%, 203,513 particles) were sorted into classes exhibiting either no additional density in the masked region, no ES27a in the exit position, or only noisy signal likely corresponding to NatB-1 around ES27a and were thus discarded.

Two of the three remaining classes (77,918 particles and 50,791 particles) showed density for a rigidly bound copy of NatB (NatB-2) at the second universal adapter site on the 60S tunnel exit and only fuzzy density for NatB-1. A third class of 115,238 particles contained density only for NatB-1, but no signal for NatB-2. All three of these classes were subjected to additional rounds of focused sorting and refinement using a mask around the expected position of NatB-1.

In this process, a majority of the particles (224,091 or 91.9% of the particles selected after initial sorting) showed a high continuous conformational heterogeneity of NatB-1 and was not processed further. One subclass of 9,645 particles, however, showed a defined density for both NatB-2 and NatB-1 in which NatB1 was positioned in direct vicinity to NatB-2 and directly below the tunnel exit. This class was termed “Class I” and refined to a resolution of 3.8 Å according to gold standard (FSC = 0.143).

All initial classes containing NatB-2 were also subjected to focused sorting on NatB-2 in a similar fashion and with the resulting class of particles with rigidly bound NatB-2 (45,530 particles), termed “Class II”, a reconstruction of the NatB2-ribosome complex at a resolution of 3.1 Å was obtained.

#### Model of the NatB-ribosome complex

The model of the ribosome was generated by adapting a model of the yeast 80S ribosome stalled on the CGA-CCG inhibitory codon combination [62] for the 60S ribosomal subunit and of ES27a in the exit position from NatA-ribosome complex [25]. A homology model of NatB (comprising Naa25 and Naa20) was obtained by using AF2 in multimer mode [37]. All models were first rigid-body fitted in ChimeraX [63] after minor adjustments, such as rearranging both ES27a and the C-terminal helices of Naa25 based on the higher-resolution reconstruction of Class II. The model for Class II, containing only NatB-2 but not NatB-1, was then refined using real space refinement in Phenix [64] and manual adjustment in WinCoot [65] using ProSmart and RCrane modules [66,67]. A model for Class I was then generated by rigid-body fitting both the model for Class II and the Alphafold model for NatB-1 into the corresponding density and performing one round of real space refinement in Phenix, followed by manual adjustments in WinCoot. All cryo-EM structures and models were displayed with ChimeraX [63].

## Supporting information

S1 Fig Affinity purification of native Map1-ribosome complexes. (**A**) Amido black stained PVDF membrane of Map1-TAP purification samples separated on a 12% Nu-PAGE gel. (**B**) ECL-developed PVDF membrane after western blotting using antibodies against the CaMBD (α-CAB) moiety of the TAP tag and against ribosomal protein uL29. A shift of the α-CAB signal upon TEV cleavage indicates the successful cleavage of the Protein A domain from the TAP tag leaving Map1-CaMBD and copurified ribosomes in the elution fraction. (**C**) 12% Nu-PAGE of the elution fraction from the Map1-ribosome purification. Ly, lysate; SN, supernatant; P, pellet; FT, flow through; R, resuspension; W, wash; E, elution; B, boiled beads. 0.1 A_260_ of E was loaded; for wash fractions, 1/17 of the volume was loaded on the gel; for Ly, SN, P, and FT, 3 μl of the sample were loaded corresponding to 1/6,000 for L, 1/8,000 for SN, and 1/1,000 for P and FT. TAP = tandem affinity purification; CaMBD = Calmodulin-binding domain. *, contamination from a viral protein. See S1 Raw Images for all raw gel and western blot images shown in (**A**, **B**, and **C**).
(TIF)

S2 Fig Classification scheme for native Map1-ribosome complexes. Particles were picked with Gautomatch followed by 2D classification in RELION to discard nonribosomal particles. Subsequent refinement and 3D classification into eight classes resulted in four high-resolution classes all showing in the exit position. These classes, comprising 86% of all particles, were joined and subjected to a masked classification on ES27 and the region around the TE. One-quarter of the particles formed a stable class with a defined ES27 and an additional density for Map1. This class was further subclassified applying a mask covering the tip of ES27 and the Map1 density. The resulting two stable classes showed Map1 in two conformations (red, blue), harboring 10% and 13% of all particles. Both classes were CTF refined to an overall resolution of 3.9 Å and 3.8 Å, respectively. All maps are shown at the same contour level; percentages refer to the previous processing step.
(TIF)

S3 Fig Local resolution and FSC curves for the Map1-C1-80S and Map1-C2-80S cryo-EM reconstructions. (**A**) Cryo-EM maps of Map1-C1-80S and Map1-C2-80S before postprocessing and colored according to local resolution as determined by RELION. Local resolution for Map1 in both maps ranged from 6.5 Å to below 12.5 Å, indicating a high degree in flexibility. (**B**) FSC curves for both refined classes; the average resolution was estimated according to the gold standard.
(TIF)

S4 Fig Comparison of Map1 bound to the ribosome tunnel exit with Map1 homologs. (**A**) Zoomed view focusing on the tunnel exit region of the Map1-C1-80S map compared to *S*.*c*. Arx1-containing pre-60S (EMD-6615), the *H*.*s*. EBP1-bound 80S (EMD-10344 and 10609) and the PDF-Map-70S ribosome complex from *E*. *coli* (EMD-9753). (**B**) Overlays of the Map1-C1-80S map with isolated densities for ES27a, Arx1, EBP1, and bacterial Map (bMap) from the maps shown above.
(TIF)

S5 Fig Fitting of the Map1 and NatB models into density. (**A**-**D**) Two views showing the fit of the Map1 AlphaFold-2 model into the isolated density from the Map1-C1 class (**A**, **B**) and Map1-C2 class (**C**, **D**). (**E**) Fit of the model for ES27a-bound NatB-1 into isolated density from Class I (Naa25-1 in rainbow, Naa20 purple, ES27a blue). (**F**) Side view of (**E**). (**G**) View showing the fit of both NatB models into the density of Class I. (**H**) Fit of the model for NatB-2 into isolated density from Class II (after focused sorting on NatB-2). Naa25-1 is shown in rainbow, Naa20 in grey. (**I**) Side view of (**H**). (**J**) View highlighting the interaction of the C-terminal α-helices of Naa25-2 (from NatB-2) with ribosomal RNA. (**H**, **I**). All maps were filtered according to local resolution.
(TIF)

S6 Fig Conformation of ES27a in Map1-, NatA-, and NatB-bound ribosomal complexes. View focusing on the exit tunnel with the position of ES27a as observed in the NatA-ribosome structure [25], in the Map1-ribosome structures (classes C1 and C2), and in the NatB-ribosome structure (class I with two stable NatBs bound) outlined. Relative rotation angles around the H63, ES27a, and ES27b three-way junction as well as the distances between the respective ES27a tip positions are shown.
(TIF)

S7 Fig NuPAGE analysis of NatB, 80S ribosomes, RNCs, and NatB mutants. 12% Nu-PAGE gels showing purified components used for the NatB project. (**A**) Lane 1, RNC_MDEL_; lane 2, Marker (PAGE Ruler Unstained, Thermo Fisher, #26614); lane 3, rt80S ribosomes; lane 4, 80S ribosomes, lane 5, empty; lane 6, NatB_wt_. (**B**) Lane 1, marker; lane 2, NatB_PP1_, lane 3, NatB_PP3_; lane 4, NatB_PP4_; lane 5 NatB_PPall_. See S1 Raw Images for all raw gel images.
(TIF)

S8 Fig Classification scheme for in vitro reconstituted NatB-RNC complex. After particle picking and 2D classification in CryoSPARC, 447,470 particles were selected for ab initio reconstruction and homogenous refinement of a consensus map. Based on 3D variability analysis of the region below the peptide exit tunnel, four principal classes were isolated. Two classes showed density for a rigidly bound copy of NatB (NatB-2) at the second universal adapter site on the 60S tunnel exit and fuzzy density for NatB-1. A third class of 115,238 particles contained density only for NatB-1, but no signal for NatB-2. All three of these classes were subjected to additional rounds of focused sorting and refinement using a mask around the expected position of NatB-1. This revealed one subclass showing a defined density for both NatB-2 and NatB-1. This subclass (class I; 9,645 particles) was refined to a resolution of 3.8 Å according to gold standard (FSC = 0.143). Other subclasses showed a high degree of conformational heterogeneity for ES27a and the bound NatBs, as exemplified by a few selected classes displayed here. In an additional, independent classification branch all initial classes containing NatB-2 were subjected to focused sorting on NatB-2 resulting in class II (45,530 particles), which was further refined to a resolution of 3.1 Å.
(TIF)

S9 Fig Local resolution and FSC curve of NatB-ribosome complexes. (**A**) Cryo-EM maps of the two main classes of the NatB-ribosome complex were low-pass filtered and colored according to local resolution in CryoSPARC. Local resolution ranged from approximately 4 Å to 9 Å for the two NatBs in class I (left) and from approximately 3 Å to 6 Å for the focused refined NatB-2 in class II (right). (**B**) FSC curves for both refined NatB-ribosome classes; the average resolution was estimated according to the gold standard to 3.8 Å and 3.1 Å, respectively.
(TIF)

S10 Fig Comparison of ribosome-bound NatB-1 and NatB-2. (**A**) Position of the NatB-1 and NatB-2 models (Naa25 grey, Naa20-1 blue, Naa20-2 red) with respect to the 60S subunit (shown as grey density). In addition, the position of acetyl-CoA (Ac-CoA) bound to each Naas20 subunit as well as putative models for the nascent chain are shown, once reaching into the catalytic center in Naa20-1 (green), once into Naa20-2 (yellow). (**B**) Same view as (**A**) but zoomed and showing only the two catalytic Naa20 subunits. (**C**, **D**) Top views of (**A**) and (**B**) (from the tunnel exit down) on the entire NatB complexes (**C**) or focusing only on the two Naa20 subunits (**D**).
(TIF)

S11 Fig Comparison of ribosome-bound NAT and Map1 complexes. (**A**, **B**) Bottom view showing an overlay of the NatA-ribosome structure (**A**) NatB-ribosome structure (**B**) with isolated densities for ribosome-bound Map1 in C1 and C2 position. (**C**) and (**D**) show side views.
(TIF)

S1 Table Cryo-EM data collection, refinement, and validation statistics for NatB-ribosome complexes. Overview over cryo-EM data collection, data processing, and model fitting parameters for the NatB-RNC_MDEL_ structures. Class I represents the data subset with two stably bound NatBs, class I the data subset focused sorted on NatB-1.
(PDF)

S1 Raw Images Raw gel and western blot images. (**A**) Raw image of 12% Nu-PAGE gel from the Map1-TAP affinity purification. The lane shown in Figs 1A and S1C is labeled with “S.” Lanes labeled with “X” were not shown or discussed in the manuscript. (**B**) Raw image of the Coomassie-stained 15% SDS-PAGE gel used in Fig 1I. Shown are samples from the Map1 co-sedimentation assay with RNCs, RNaseI-treated RNCs (rtRNCs), and nonprogrammed ribosomes (np80S). Lanes labeled with “X” were not shown or discussed in the manuscript. (**C**) Raw images of the western blot from the first replicate of NatB binding assay using wild type (WT) NatB and PP1, PP4, and PP_all NatB mutants. Top: Chemiluminescence image; levels were adjusted for optimum contrast. This image was also used in the quantification of NatB binding shown in Fig 2I (see also S1 Data). Bottom: Overlay of adjusted chemiluminescence image and visible light image for marker. Lanes labeled with “X” were not used for quantification. (**D**) Raw images of the western blot from the second replicate of NatB binding assay using wild type (WT) NatB and PP1, PP4, and PP_all NatB mutants. Top: Chemiluminescence image; levels were adjusted for optimum contrast. This image was also used in the quantification of NatB binding shown in Fig 2I (see also S1 Data) and is displayed as representative western blot in Fig 2I. Bottom: Overlay of adjusted chemiluminescence image and visible light image for marker. Lanes labeled with “S” were shown as representative signals in Fig 2I. Lanes with “X” were not used for quantification or shown in the manuscript. (**E**) Raw images of the western blot from the third replicate of NatB binding assay using wild type (WT) NatB and PP1, PP4, and PP_all NatB mutants. Top: Chemiluminescence image; levels were adjusted for optimum contrast. This image was also used in the quantification of NatB binding shown in Fig 2I (see also S1 Data). Bottom: Overlay of adjusted chemiluminescence image and visible light image for marker. Lanes labeled with “X” were not used for quantification. (**F**) Raw images of the western blot of NatB binding assay using wild type (WT) NatB and the PP3 NatB mutant. Top: Chemiluminescence image; levels were adjusted for optimum contrast. This image was also used in the quantification of NatB binding shown in Fig 2I (see also S1 Data). Bottom: Overlay of adjusted chemiluminescence image and visible light image for marker. Lanes labeled with “X” were not used for quantification. (**G**) Raw images of the western blot of NatB binding assay using idle ribosomes (80S) or RNaseI-treated idle ribosomes (rt80S). Top: Chemiluminescence image; levels were adjusted for optimum contrast. This image was also used in the quantification of NatB binding shown in Fig 2I (see also S1 Data). Bottom: Overlay of adjusted chemiluminescence image and visible light image for marker. Lanes labeled with “S” were shown as representative signals in Fig 2I. Lanes labeled with “X” were not used for quantification or shown in the manuscript. (**H**) Raw image of PVDF membrane stained with Amido black from western blot of affinity purification of native Map1-ribosome complexes as used in S1A Fig. (**I**) Raw chemiluminescence images from western blot of affinity purification of native Map1-ribosome complexes as shown in S1B Fig. Top: raw image after incubation with anti-CAB antibody. Bottom: raw image after incubation with anti-uL29 antibody. Levels were uniformly adjusted for optimal contrast. (**J**) Raw image of Coomassie-stained 12% NuPAGE gel showing input samples used for NatB binding assays (Fig 2I). Wild type NatB (NatB_WT_), RNCs, RNaseI-treated ribosomes (rt80S), and untreated ribosomes (80S) are labeled and were used in S7A Fig. Lanes labeled with “X” were not shown or discussed in the manuscript. (**K**) Raw image of the Coomassie-stained 12% NuPAGE gel showing purified NatB positive-patch mutants. Lanes shown in S7B Fig are labeled. Lanes labeled with “X” were not shown or discussed in the manuscript.
(PDF)

S1 Data Numerical data for densitometric quantification. Numerical data obtained from densitometric quantification of gel and western blot images using ImageJ version 1.53Q and calculations of averages and errors shown in Figs 1I and 2I. For Fig 1I, band intensities relative to background of the Map1 band in the Coomassie-stained gel were determined and normalized to the control with untreated RNCs. For Fig 2I, band intensities over background for bands corresponding to Naa25 and uL29 were determined for supernatant and pellet fraction, and the ratio of the Naa25 band intensities was calculated for each pair of fractions. Ratios were normalized by the band intensity measured for the ribosomal protein in each corresponding pellet fraction. Averages of these ratios were determined from the replicates and normalized to the wild type control experiment, where the binding efficiency in the control was set to 100%. Errors were calculated as standard deviations of the averages determined from replicates.
(XLSX)

## Abbreviations

acetyl-CoA: acetyl-coenzyme A
AF2: AlphaFold 2
APD: amino peptidase domain
CTF: contrast transfer function
ES: expansion segment
MetAP: methionine aminopeptidase
NAC: nascent polypeptide-associated complex
NAT: N-acetyl-transferase
PDF: peptidyl-deformylase
RAC: ribosome-associated complex
RNC: ribosome nascent chain complex
rtRNC: RNaseI-treated RNC
SN: supernatant
SRP: signal recognition particle
TAP: tandem affinity purification
TEV: tobacco etch virus
TPR: tetratricopeptide repeat
TRAP: Translocon Associated Protein
wt: wild type
