# Supplementary figures and images for: The dynamic architecture of Map1- and NatB-ribosome complexes coordinates the sequential modifications of nascent polypeptide chains
Source: PLoS Biol. 2023 Apr 20;21(4):e3001995. doi: 10.1371/journal.pbio.3001995 (PMC10118133; doi:10.1371/journal.pbio.3001995)

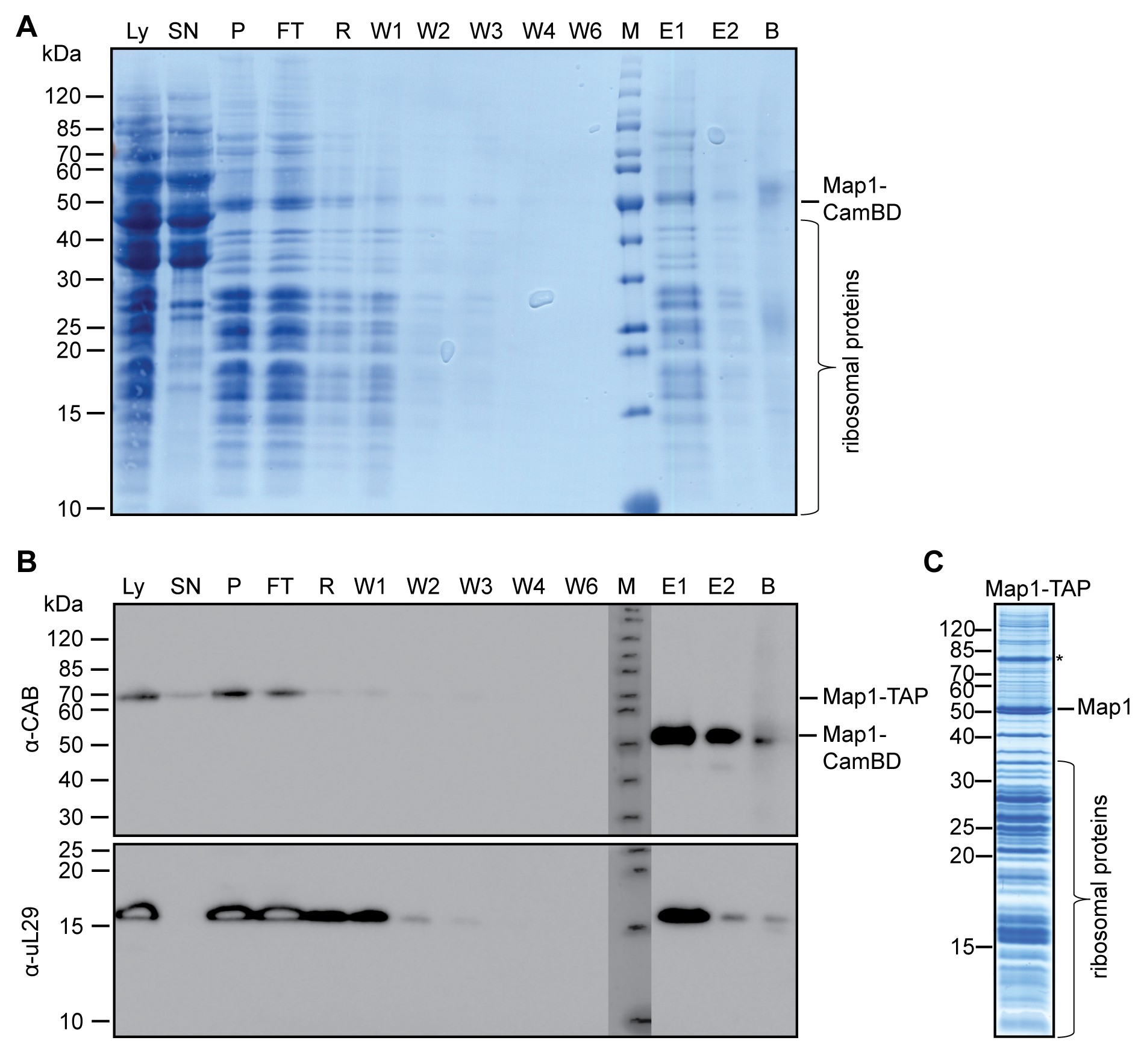

Supplement: S1 Fig — (A) Amido black stained PVDF membrane of Map1-TAP purification samples separated on a 12% Nu-PAGE gel. (B) ECL-developed PVDF membrane after western blotting using antibodies against the CaMBD (α-CAB) moiety of the TAP tag and against ribosomal protein uL29. A shift of the α-CAB signal upon TEV cleavage indicates the successful cleavage of the Protein A domain from the TAP tag leaving Map1-CaMBD and copurified ribosomes in the elution fraction. (C) 12% Nu-PAGE of the elution fraction from the Map1-ribosome purification. Ly, lysate; SN, supernatant; P, pellet; FT, flow through; R, resuspension; W, wash; E, elution; B, boiled beads. 0.1 A260 of E was loaded; for wash fractions, 1/17 of the volume was loaded on the gel; for Ly, SN, P, and FT, 3 μl of the sample were loaded corresponding to 1/6,000 for L, 1/8,000 for SN, and 1/1,000 for P and FT. TAP = tandem affinity purification; CaMBD = Calmodulin-binding domain. *, contamination from a viral protein. See S1 Raw Images for all raw gel and western blot images shown in (A, B, and C). (TIF) [file pbio.3001995.s001.tif]

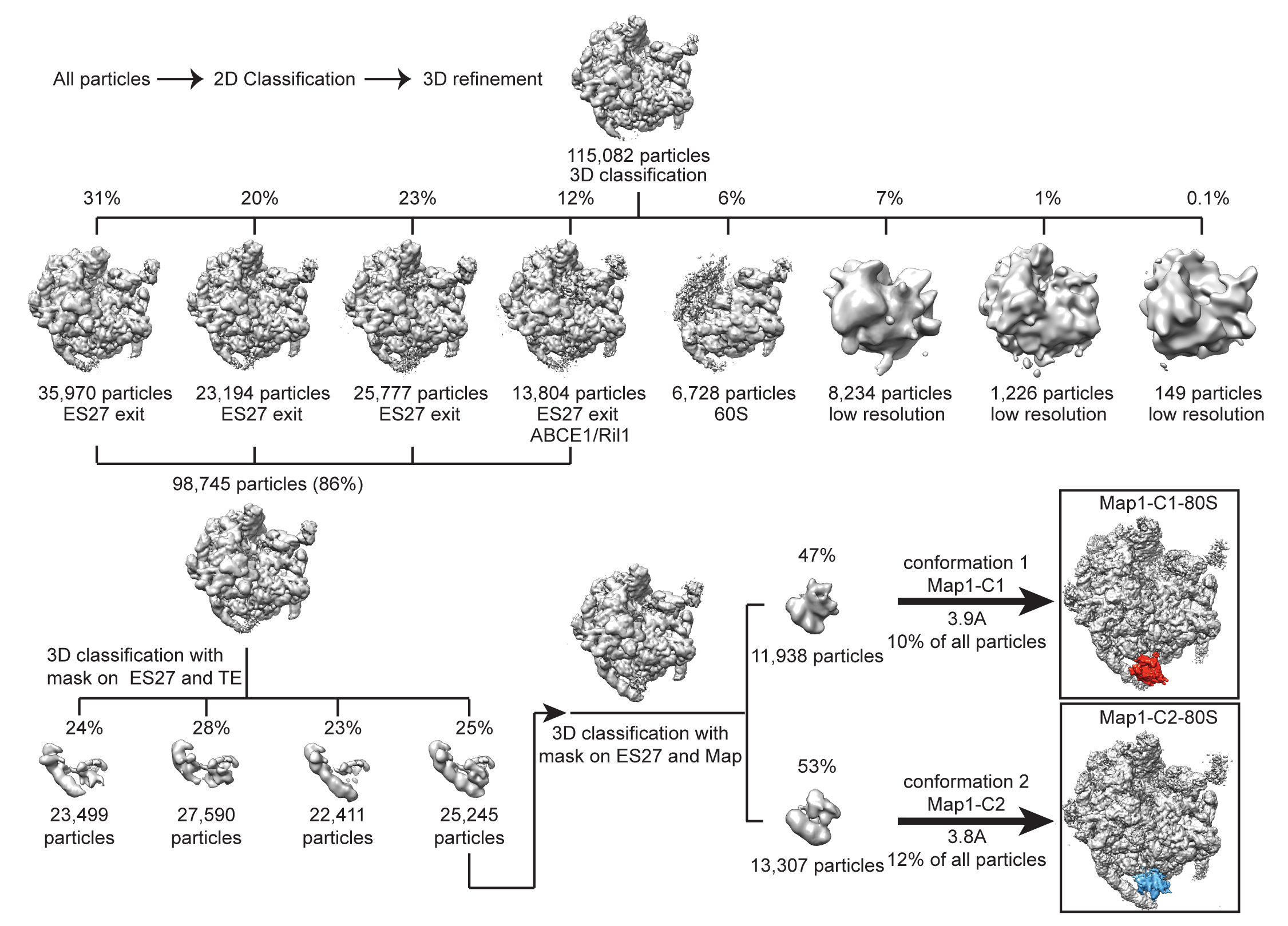

Supplement: S2 Fig — Particles were picked with Gautomatch followed by 2D classification in RELION to discard nonribosomal particles. Subsequent refinement and 3D classification into eight classes resulted in four high-resolution classes all showing in the exit position. These classes, comprising 86% of all particles, were joined and subjected to a masked classification on ES27 and the region around the TE. One-quarter of the particles formed a stable class with a defined ES27 and an additional density for Map1. This class was further subclassified applying a mask covering the tip of ES27 and the Map1 density. The resulting two stable classes showed Map1 in two conformations (red, blue), harboring 10% and 13% of all particles. Both classes were CTF refined to an overall resolution of 3.9 Å and 3.8 Å, respectively. All maps are shown at the same contour level; percentages refer to the previous processing step. (TIF) [file pbio.3001995.s002.tif]

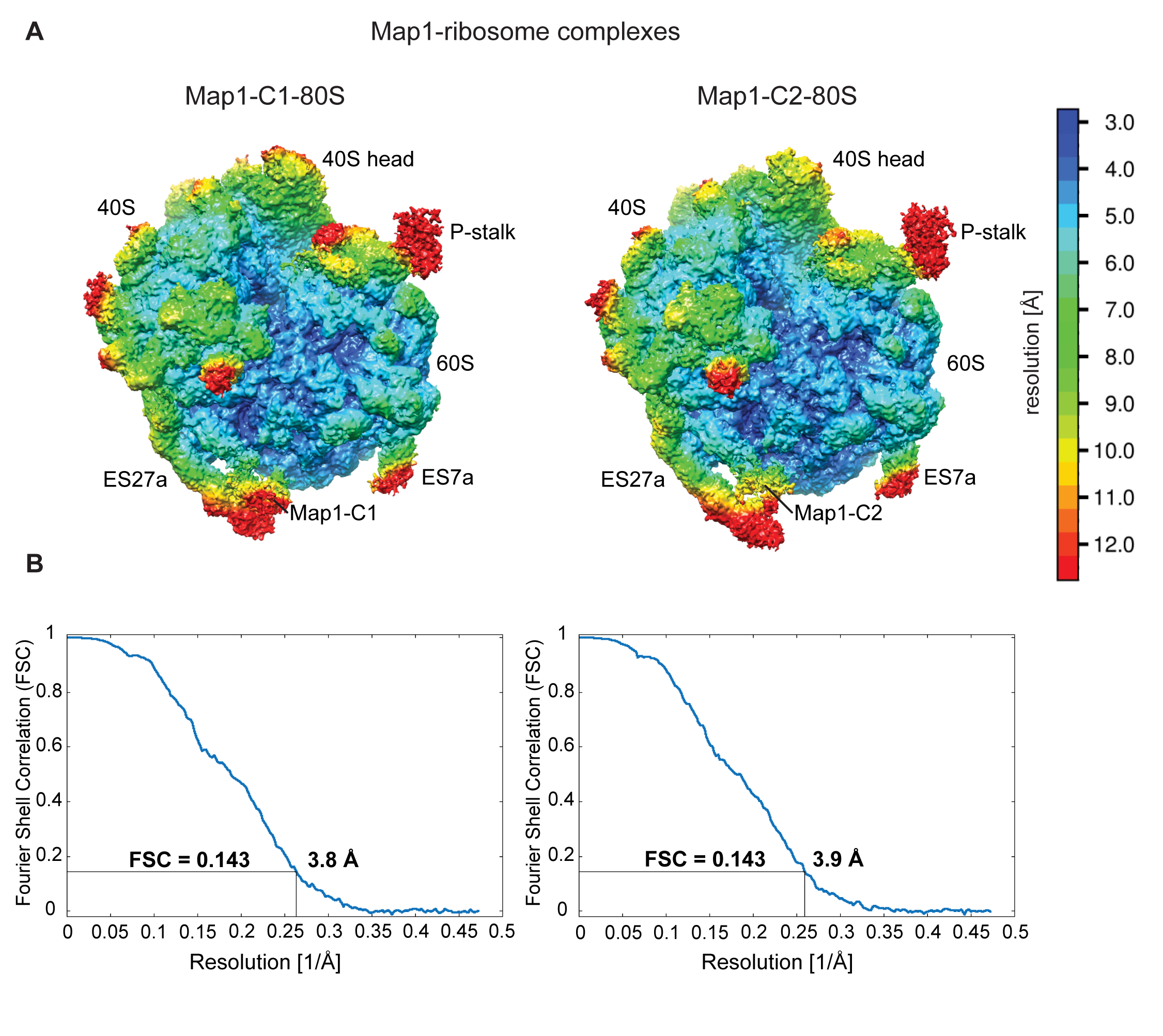

Supplement: S3 Fig — (A) Cryo-EM maps of Map1-C1-80S and Map1-C2-80S before postprocessing and colored according to local resolution as determined by RELION. Local resolution for Map1 in both maps ranged from 6.5 Å to below 12.5 Å, indicating a high degree in flexibility. (B) FSC curves for both refined classes; the average resolution was estimated according to the gold standard. (TIF) [file pbio.3001995.s003.tif]

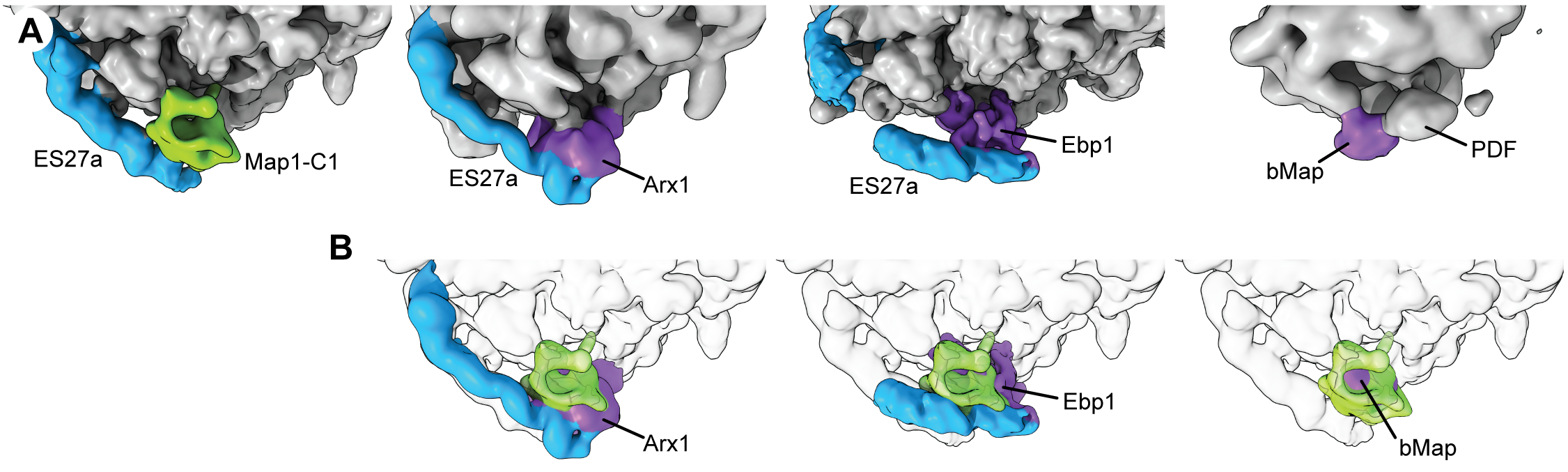

Supplement: S4 Fig — (A) Zoomed view focusing on the tunnel exit region of the Map1-C1-80S map compared to S.c. Arx1-containing pre-60S (EMD-6615), the H.s. EBP1-bound 80S (EMD-10344 and 10609) and the PDF-Map-70S ribosome complex from E. coli (EMD-9753). (B) Overlays of the Map1-C1-80S map with isolated densities for ES27a, Arx1, EBP1, and bacterial Map (bMap) from the maps shown above. (TIF) [file pbio.3001995.s004.tif]

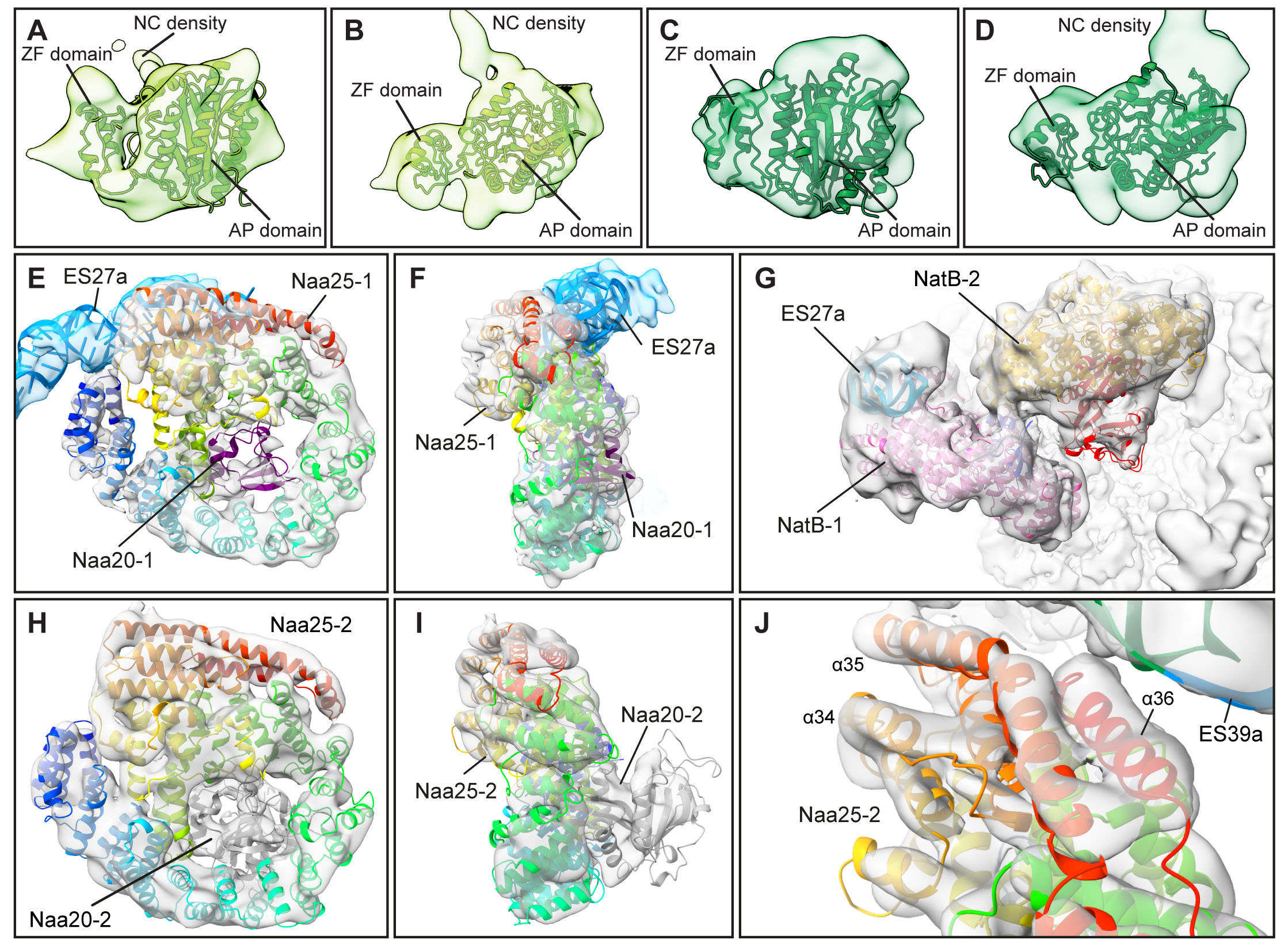

Supplement: S5 Fig — (A-D) Two views showing the fit of the Map1 AlphaFold-2 model into the isolated density from the Map1-C1 class (A, B) and Map1-C2 class (C, D). (E) Fit of the model for ES27a-bound NatB-1 into isolated density from Class I (Naa25-1 in rainbow, Naa20 purple, ES27a blue). (F) Side view of (E). (G) View showing the fit of both NatB models into the density of Class I. (H) Fit of the model for NatB-2 into isolated density from Class II (after focused sorting on NatB-2). Naa25-1 is shown in rainbow, Naa20 in grey. (I) Side view of (H). (J) View highlighting the interaction of the C-terminal α-helices of Naa25-2 (from NatB-2) with ribosomal RNA. (H, I). All maps were filtered according to local resolution. (TIF) [file pbio.3001995.s005.tif]

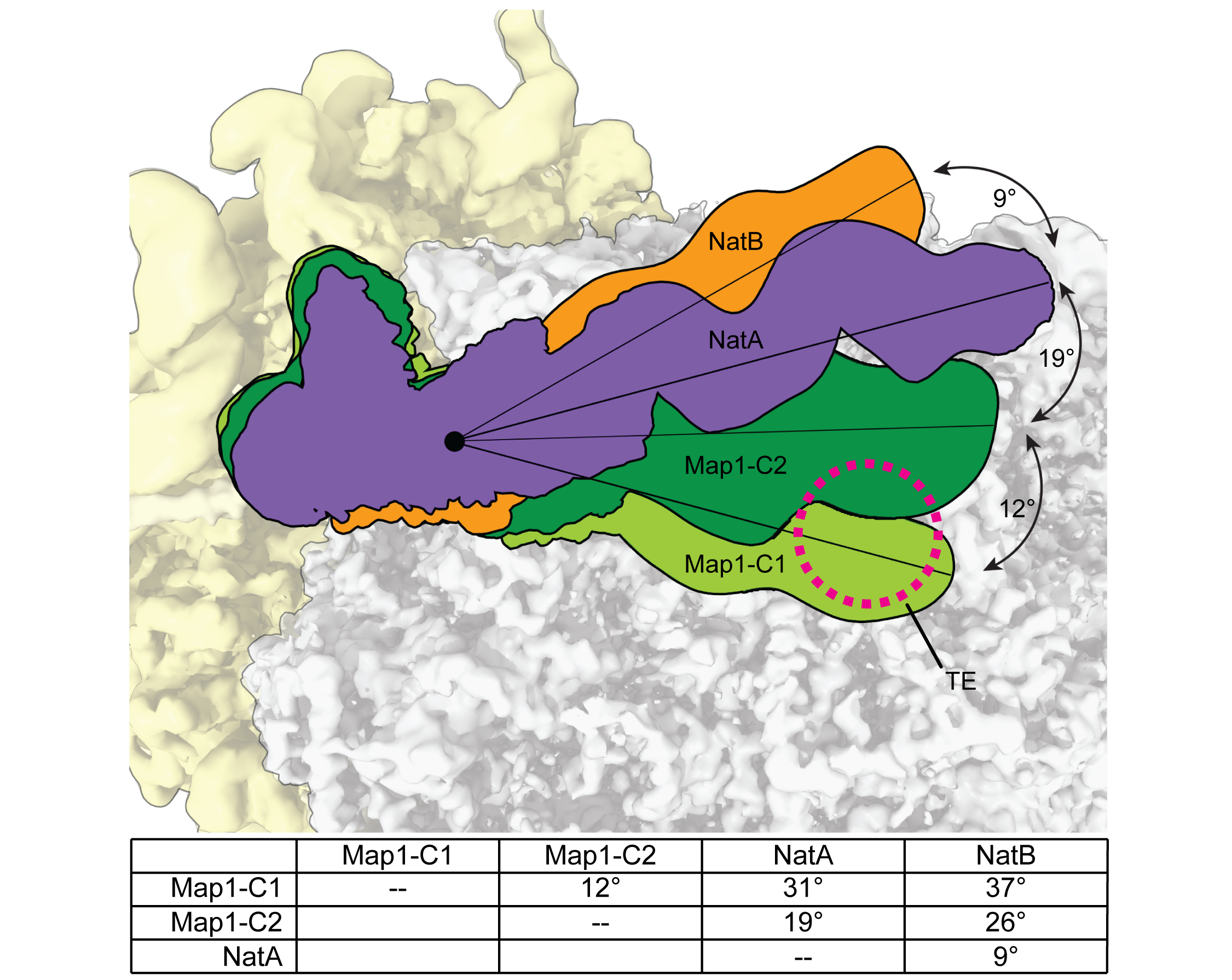

Supplement: S6 Fig — View focusing on the exit tunnel with the position of ES27a as observed in the NatA-ribosome structure [25], in the Map1-ribosome structures (classes C1 and C2), and in the NatB-ribosome structure (class I with two stable NatBs bound) outlined. Relative rotation angles around the H63, ES27a, and ES27b three-way junction as well as the distances between the respective ES27a tip positions are shown. (TIF) [file pbio.3001995.s006.tif]

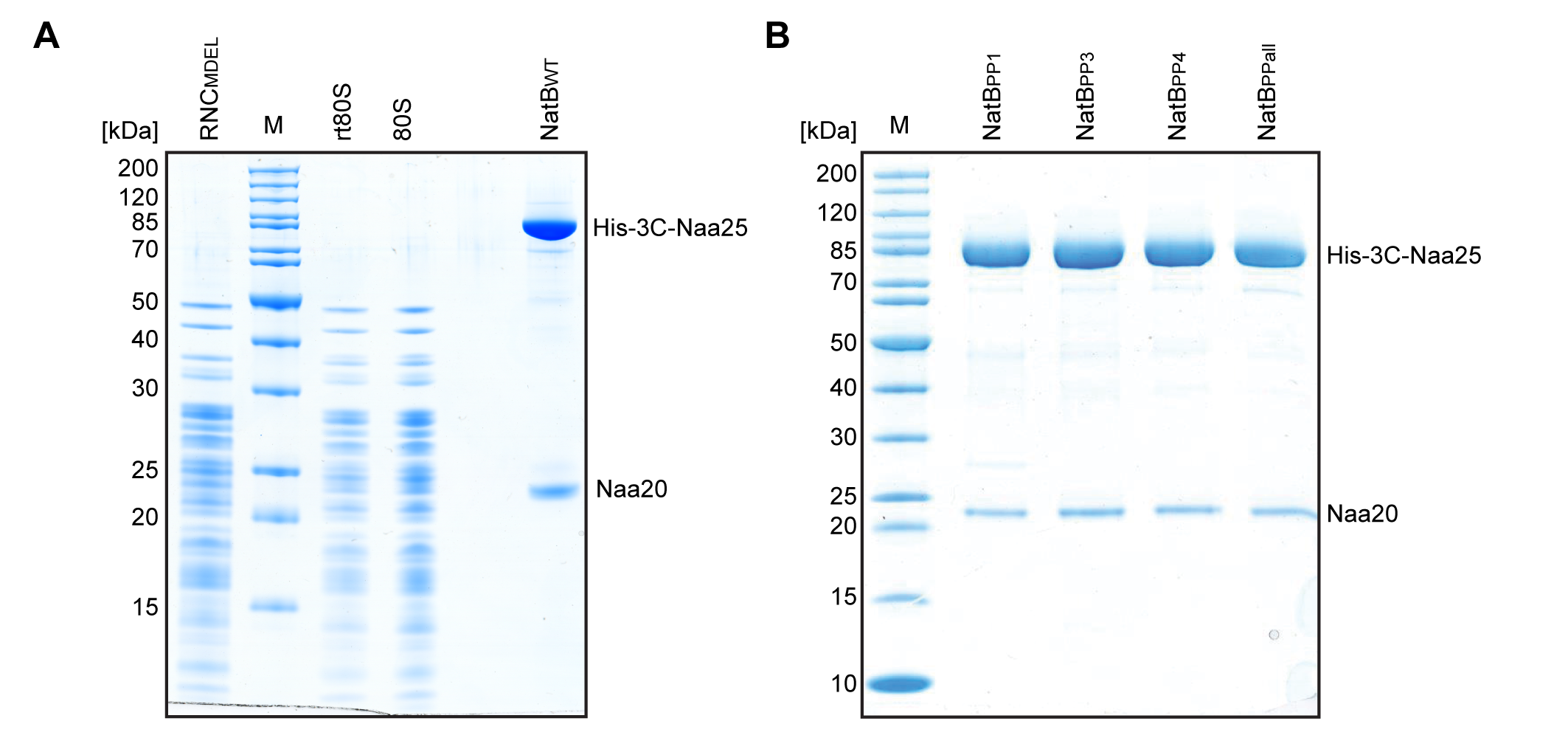

Supplement: S7 Fig — 12% Nu-PAGE gels showing purified components used for the NatB project. (A) Lane 1, RNCMDEL; lane 2, Marker (PAGE Ruler Unstained, Thermo Fisher, #26614); lane 3, rt80S ribosomes; lane 4, 80S ribosomes, lane 5, empty; lane 6, NatBwt. (B) Lane 1, marker; lane 2, NatBPP1, lane 3, NatBPP3; lane 4, NatBPP4; lane 5 NatBPPall. See S1 Raw Images for all raw gel images. (TIF) [file pbio.3001995.s007.tif]

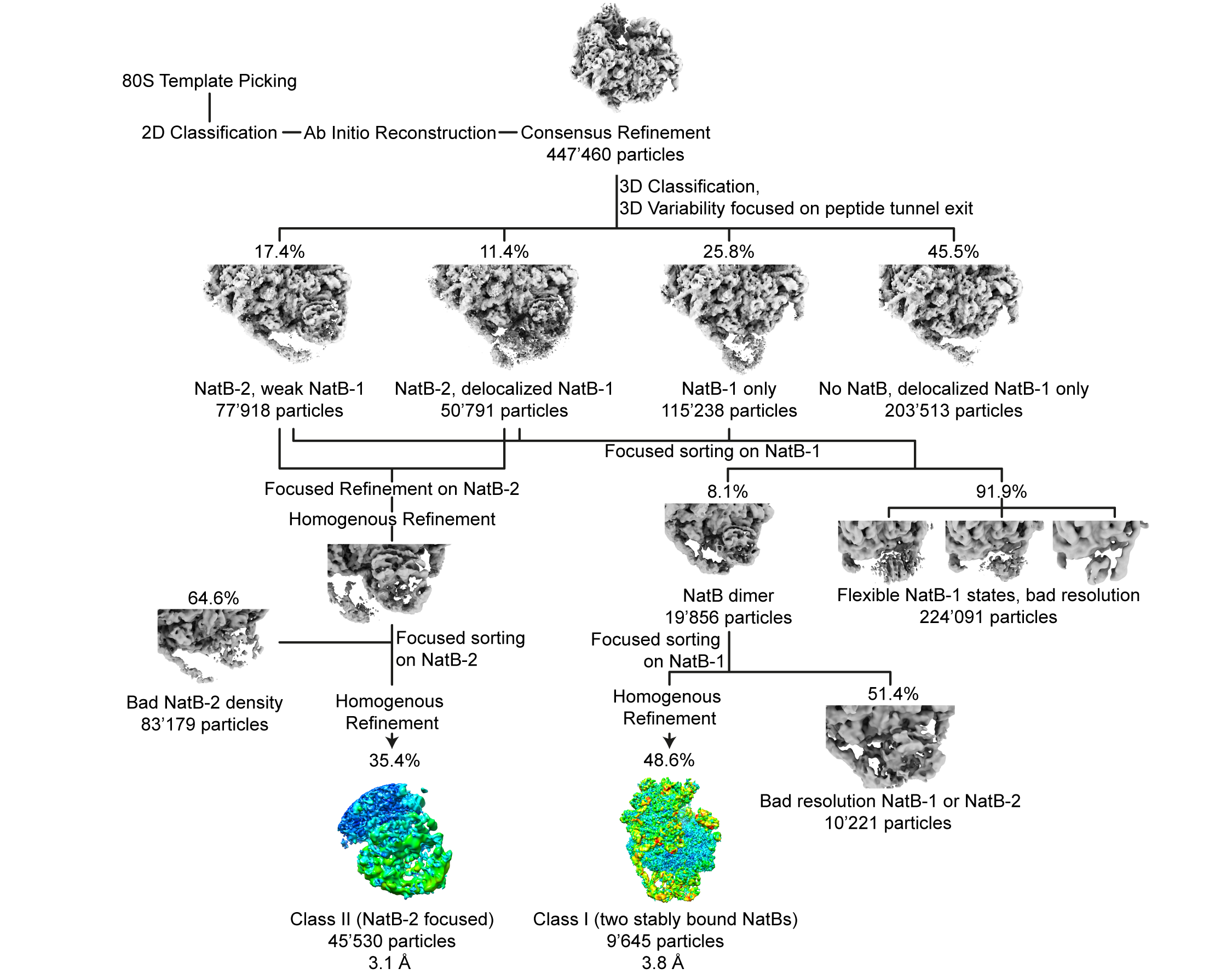

Supplement: S8 Fig — After particle picking and 2D classification in CryoSPARC, 447,470 particles were selected for ab initio reconstruction and homogenous refinement of a consensus map. Based on 3D variability analysis of the region below the peptide exit tunnel, four principal classes were isolated. Two classes showed density for a rigidly bound copy of NatB (NatB-2) at the second universal adapter site on the 60S tunnel exit and fuzzy density for NatB-1. A third class of 115,238 particles contained density only for NatB-1, but no signal for NatB-2. All three of these classes were subjected to additional rounds of focused sorting and refinement using a mask around the expected position of NatB-1. This revealed one subclass showing a defined density for both NatB-2 and NatB-1. This subclass (class I; 9,645 particles) was refined to a resolution of 3.8 Å according to gold standard (FSC = 0.143). Other subclasses showed a high degree of conformational heterogeneity for ES27a and the bound NatBs, as exemplified by a few selected classes displayed here. In an additional, independent classification branch all initial classes containing NatB-2 were subjected to focused sorting on NatB-2 resulting in class II (45,530 particles), which was further refined to a resolution of 3.1 Å. (TIF) [file pbio.3001995.s008.tif]

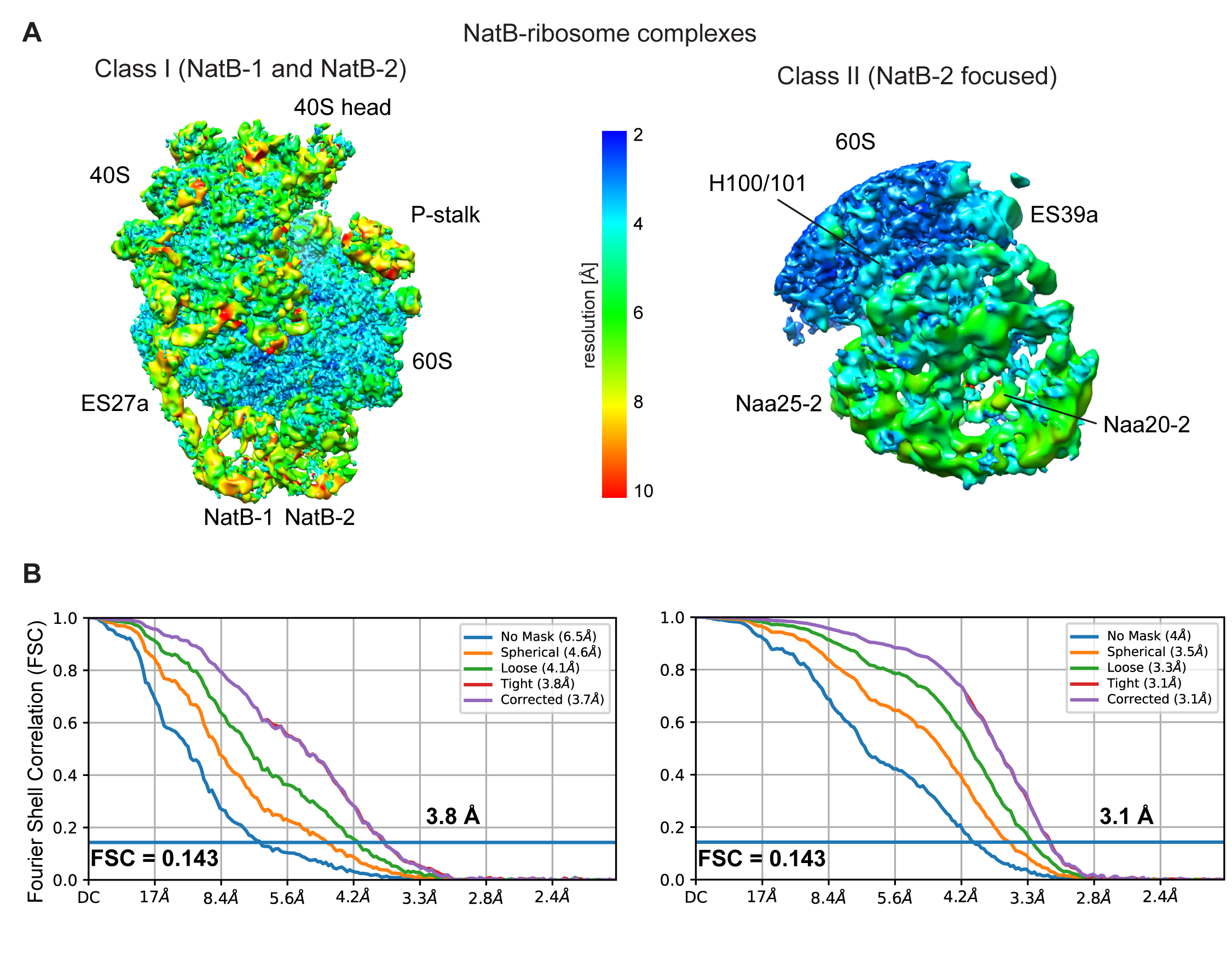

Supplement: S9 Fig — (A) Cryo-EM maps of the two main classes of the NatB-ribosome complex were low-pass filtered and colored according to local resolution in CryoSPARC. Local resolution ranged from approximately 4 Å to 9 Å for the two NatBs in class I (left) and from approximately 3 Å to 6 Å for the focused refined NatB-2 in class II (right). (B) FSC curves for both refined NatB-ribosome classes; the average resolution was estimated according to the gold standard to 3.8 Å and 3.1 Å, respectively. (TIF) [file pbio.3001995.s009.tif]

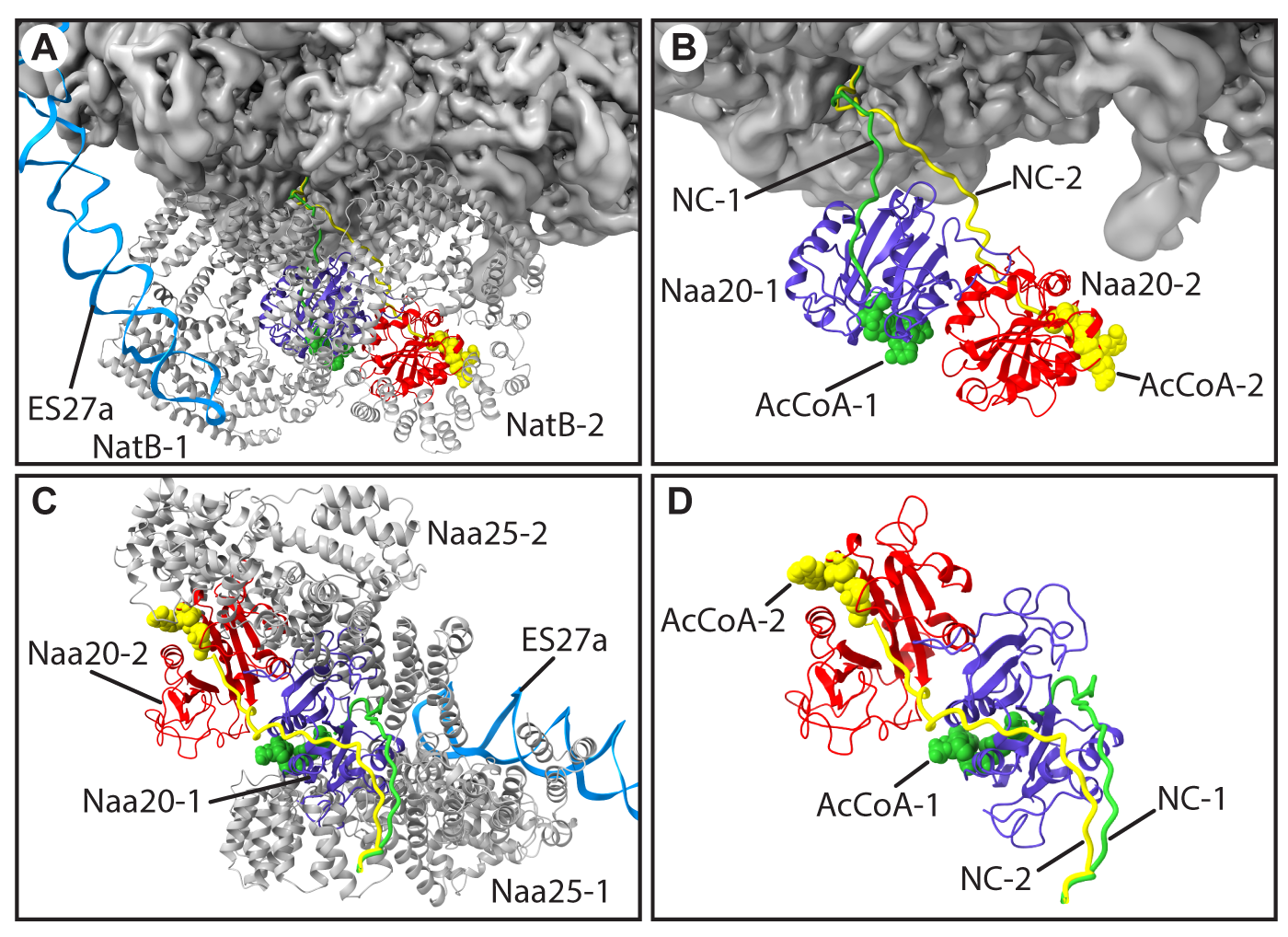

Supplement: S10 Fig — (A) Position of the NatB-1 and NatB-2 models (Naa25 grey, Naa20-1 blue, Naa20-2 red) with respect to the 60S subunit (shown as grey density). In addition, the position of acetyl-CoA (Ac-CoA) bound to each Naas20 subunit as well as putative models for the nascent chain are shown, once reaching into the catalytic center in Naa20-1 (green), once into Naa20-2 (yellow). (B) Same view as (A) but zoomed and showing only the two catalytic Naa20 subunits. (C, D) Top views of (A) and (B) (from the tunnel exit down) on the entire NatB complexes (C) or focusing only on the two Naa20 subunits (D). (TIF) [file pbio.3001995.s010.tif]

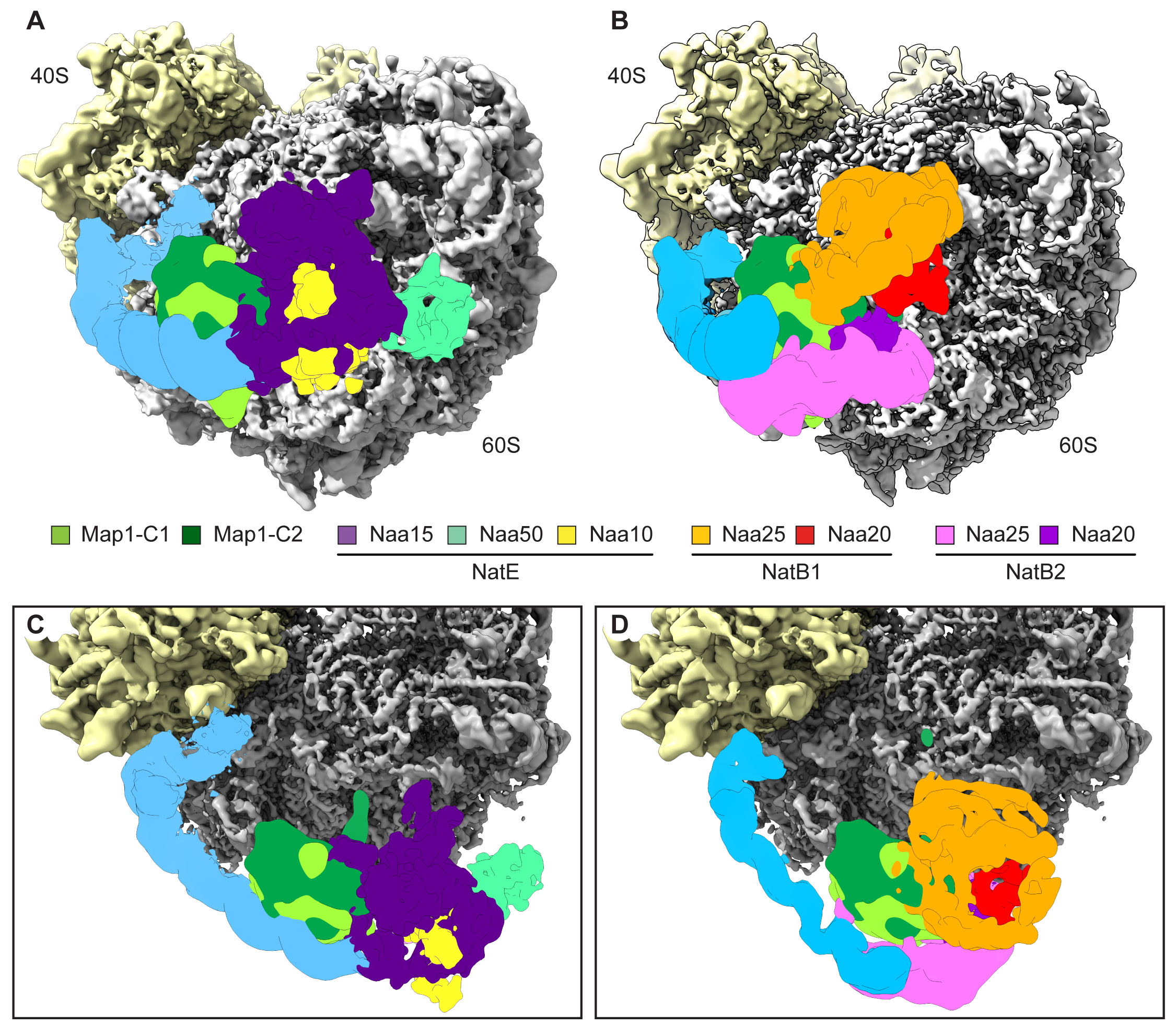

Supplement: S11 Fig — (A, B) Bottom view showing an overlay of the NatA-ribosome structure (A) NatB-ribosome structure (B) with isolated densities for ribosome-bound Map1 in C1 and C2 position. (C) and (D) show side views. (TIF) [file pbio.3001995.s011.tif]
