# Supplementary material for: The dynamic architecture of Map1- and NatB-ribosome complexes coordinates the sequential modifications of nascent polypeptide chains
Source: PLoS Biol. 2023 Apr 20;21(4):e3001995. doi: 10.1371/journal.pbio.3001995 (PMC10118133; doi:10.1371/journal.pbio.3001995)
Supplement: S1 Table — Overview over cryo-EM data collection, data processing, and model fitting parameters for the NatB-RNCMDEL structures. Class I represents the data subset with two stably bound NatBs, class I the data subset focused sorted on NatB-1. (PDF) [file pbio.3001995.s012.pdf]

	Class I ***NatB-RNC$_{MDEL}$ complex with two stably bound NatBs***	***Class II*** ***NatB-RNC$_{MDEL}$ focused sorted on NatB-2***
PDB	8BJQ	8BIP
EMDB	EMD-16090	EMD-16086
**Data collection and processing**		
Microscope	FEI Titan Krios	
Voltage (kV)	300	
Camera	Gatan K2 Summit 4kx4k	
Magnification	130.000	
Nominal defocus range (µm)	0.5-3.5	
Electron exposure (e–/Å²)	45.2	
Number of frames	40	
Pixel size (Å)	1.045	
Micrographs	10,380	
**Refinement**		
Particles in class	9,645	45,530
Map resolution (FSC 0.143) (Å)	3.8	3.1
Map sharpening B factor	-46.2	-70.3
Map vs. model CC	0.79	0.89
**Model composition**		
Non-hydrogen atoms	141437	134330
Protein residues	8092	7228
Nucleotide residues	3580	3580
Water	0	0
Ligands	ZN:5 MG:206	ZN:5 MG:206
**B factors (Å²)**		
Protein	180.89	199.43
Nucleotide	142.21	158.85
Ligand	104.69	99.15
**R.m.s. deviations**		
Bond lengths	0.005	0.003
Bond angles	0.685	0.528
**Validation**		
MolProbity Score	1.90	1.89
All-atom clash score	11.56	10.76
Poor rotamers (%)	0.07	0.03
**Ramachandran plot**		
Favored (%)	95.44	95.15
Allowed (%)	4.43	4.75
Outlier (%)	0.14	0.10
